# Deep Learning Artificial Intelligence and Restriction Spectrum Imaging for Patient-level Detection of Clinically Significant Prostate Cancer on Biparametric Magnetic Resonance Imaging

**DOI:** 10.1016/j.euros.2026.01.014

**Published:** 2026-02-06

**Authors:** Yuze Song, Mariluz Rojo Domingo, Christopher C. Conlin, Deondre D. Do, Madison T. Baxter, Anna Dornisch, George Xu, Aditya Bagrodia, Tristan Barrett, Mukesh Harisinghani, Gary Hollenberg, Sophia Kamran, Christopher J. Kane, Dimitri A. Kessler, Joshua Kuperman, Kanglung Lee, Michael A. Liss, Daniel J.A. Margolis, Paul M. Murphy, Nabih Nakrour, Truong Ngyuen, Thomas L. Osinski, Rebecca Rakow-Penner, Shoumik Roychowdhury, Ahmed S. Shabik, Shaun Trecarten, Natasha Wehrli, Eric P. Weinberg, Sean A. Woolen, Anders M. Dale, Tyler M. Seibert

**Affiliations:** a Department of Radiation Medicine, University of California-San Diego, La Jolla, CA, USA; b Department of Electrical and Computer Engineering, University of California-San Diego, La Jolla, CA, USA; c Department of Radiology, University of California-San Diego, La Jolla, CA, USA; d Department of Bioengineering, University of California-San Diego, La Jolla, CA, USA; e Department of Urology, University of California-San Diego, La Jolla, CA, USA; f Department of Radiology, Cambridge University, Cambridge, UK; g Department of Radiology, Massachusetts General Hospital, Boston, MA, USA; h Department of Clinical Imaging Sciences, University of Rochester Medical Center, Rochester, NY, USA; i Department of Radiation Oncology, Massachusetts General Hospital, Boston, MA, USA; j Artificial Intelligence in Medicine Laboratory, Universitat de Barcelona, Barcelona, Spain; k Department of Radiology, Cornell University, Ithaca, NY, USA; l Department of Urology, University of Rochester Medical Center, Rochester, NY, USA; m Department of Electrical Engineering and Computer Sciences, University of California-Berkeley, Berkeley, CA, USA; n Department of Pathology, University of California-San Diego, La Jolla, CA, USA; o Department of Urology, University of Texas Health Sciences Center, San Antonio, TX, USA; p Department of Radiology and Biomedical Imaging, University of California-San Francisco, San Francisco, CA, USA; q Department of Neurosciences, University of California-San Diego, La Jolla, CA, USA; r Halıcıoğlu Data Science Institute, University of California-San Diego, La Jolla, CA, USA

**Keywords:** Prostate cancer, Magnetic resonance imaging, Deep learning

## Abstract

**Background and objective:**

Our aim was to evaluate whether combining the maximum restriction score derived from restriction spectrum imaging (RSIrs_max_) with deep learning (DL) models can enhance patient-level detection of clinically significant prostate cancer (csPCa) in comparison to Prostate Imaging-Reporting and Data System (PI-RADS) or RSIrs_max_ alone.

**Methods:**

A total of 1892 patients from seven institutions who underwent imaging between January 2016 and March 2024 were included on the basis of magnetic resonance imaging (MRI) findings and biopsy-confirmed prostate cancer diagnosis. Two DL architectures, 3D-DenseNet and 3D-DenseNet+RSI (incorporating RSIrs_max_), were developed and trained using biparametric MRI and RSI data using a leave-one-center-out validation approach. RSI is a rapid sequence that requires only 2–3 min to acquire. Model performance was evaluated in a biopsy-confirmed subset of 876 patients, with subgroup analyses stratified by site and scanner vendor. Receiver operating characteristic (ROC) and precision recall curves and forest plots (I^2^ for heterogeneity) were generated, and the area under the ROC curve (AUC) and sensitivity, were compared, as well as specificity at fixed sensitivity of 0.90. Calibration, decision-curve, and reclassification analyses (net reclassification improvement and integrated discrimination improvement) were performed. Codes used in developing the DL model are available on GitHub (https://github.com/ESONG1999/Deep-learning-AI-and-RSI-for-patient-level-detection-of-csPCa-on-MRI).

**Key findings and limitations:**

Neither RSIrs_max_ nor the best DL model combined with RSIrs_max_ significantly outperformed PI-RADS interpretation by expert radiologists. However, when combined with PI-RADS, both approaches significantly improved patient-level csPCa detection, with AUCs of 0.78 (95% confidence interval [CI] 0.75–0.81; *p* < 0.001) for RSIrs_max_ + PI-RADS and 0.80 (95% CI 0.77–0.82; *p* < 0.001) for the best DL model + PI-RADS, versus 0.75 (95% CI 0.71–0.78) for PI-RADS alone. The absolute gain in specificity at fixed sensitivity of 0.90 was 0.04 (95% CI 0.04–0.04) for RSIrs_max_ + PI-RADS, and 0.03 (95% CI 0.03–0.04) for DL + PI-RADS.

**Conclusions and clinical implications:**

Both RSIrs_max_ and the best DL model demonstrated comparable performance to PI-RADS alone. Addition of either model to PI-RADS significantly enhanced patient-level detection of csPCa in comparison to PI-RADS alone. Limitations include biopsy as an imperfect reference, the exclusion of hip implant cases, lack of external calibration, limited RSI availability, and missing case-level information for individual radiologists and their expertise.

**Patient summary:**

We looked at whether adding advanced scan data (ASD) and artificial intelligence (AI) models to radiologist assessments of MRI (magnetic resonance imaging) scans was better in detecting aggressive prostate cancer (PCa). We found that adding AI models or ASD to standard scan scores improved cancer detection in comparison to standard scores alone. The results suggest that combining radiologist expertise with AI and ASD may help in earlier identification of more patients with csPCa.

## Take Home Message

Combining restriction scores derived from advanced restriction spectrum imaging (RSIrs) with deep learning (DL) offers a complementary advantage to expert Prostate Imaging-Reporting and Data System (PI-RADS) assessment. While neither the maximum RSIrs nor a DL model alone surpassed radiologist PI-RADS performance, integration of either approach with PI-RADS significantly improved patient-level detection of clinically significant prostate cancer. These findings suggest that quantitative imaging and artificial intelligence tools can enhance, rather than replace, expert interpretation in magnetic resonance imaging for prostate cancer diagnosis.

## 1 Introduction

Multiparametric magnetic resonance imaging (mpMRI) plays a key role in early diagnosis of prostate cancer (PCa), as recommended by the European Association of Urology (EAU) and National Comprehensive Cancer Network guidelines [1], [2]. It has been shown that mpMRI can reduce unnecessary biopsies and improve the detection of clinically significant PCa (csPCa, grade group (GG) ≥2) [1], [2], [3], [4]. The Prostate Imaging-Reporting and Data System (PI-RADS) was developed to provide a standardized approach for mpMRI interpretation [5]. However, interpretation can still vary according to the reader’s experience and training. As PCa incidence is expected to increase in the coming years, there may be challenges in meeting demand with the current supply of trained experts [6]. An accurate, supportive tool for interpretation of prostate MRI could facilitate standardization and address variability in clinical practice [7]. The strongest evidence available for diagnostic prostate MRI in current clinical practice is for patient-level detection of csPCa, especially in guiding decisions on which patients need to undergo biopsy [8], [9], [10], [11]. The most important clinical question, at least initially, is whether a patient has csPCa, with MRI use for this purpose endorsed in major guidelines [1], [12]. The goal of the present study was to address automated detection of csPCa on MRI without reliance on subjective lesion identification by radiologists, which is highly dependent on reader expertise. In a retrospective study, targeted biopsy information was only available for lesions identified by a radiologist before the procedure, and even those results are subject to the uncertainty of needle placement [13]. Thus, while lesion-level analysis is also important, we focus here on patient-level results [14], [15], [16].

Restriction spectrum imaging (RSI) is an advanced technique for diffusion-weighted imaging (DWI) that measures the signal from four distinct tissue compartments: restricted intracellular water (RSI-C_1_), hindered extracellular water (RSI-C_2_), freely diffusing water (RSI-C_3_), and vascular flow (RSI-C_4_) [17], [18]. The RSI restriction score (RSIrs) is a quantitative biomarker that is superior to the MRI apparent diffusion coefficient (ADC) for csPCa detection [14], [16], [19], [20]. Moreover, when the maximum RSIrs (RSIrs_max_) was combined with PI-RADS, the performance for patient-level csPCa detection was superior to either approach alone [14], [16].

PI-RADS relies predominantly on the T2-weighted (T2W) imaging and DWI components of mpMRI, collectively called biparametric MRI (bpMRI) [5]. There is interest in moving towards bpMRI for many patients, as bpMRI avoids the risks and costs associated with intravenous contrast [21], [22], [23], [24], [25]. Deep learning (DL) artificial intelligence (AI) models based on bpMRI have been developed for objective and reproducible detection and localization of csPCa, with results matching those of expert radiologists [26], [27], [28], [29], [30]. However, most AI models are limited by the conventional diffusion signal, which may under-represent the microstructural detail captured by RSI. Integration of RSI with AI may therefore provide an incremental biological signal beyond standard bpMRI, although differences in acquisition and reconstruction protocols across centers pose harmonization challenges for widespread adoption.

Our prespecified primary hypothesis was that combining RSI with bpMRI-based DL AI, or integration of both with PI-RADS, would improve patient-level detection of csPCa in comparison to either approach alone. The analysis plan included a multi-institutional evaluation with site-level clustering, predefined operating threshold of fixed sensitivity of 0.90, and 10 000 bootstraps to estimate confidence intervals (CIs) and assess generalizability. Model performance was evaluated on a biopsy-confirmed subset of 876 patients across seven institutions, with subgroup analyses stratified by site and scanner vendor. Results for the area under the receiver operating characteristic (ROC) curve (AUC), sensitivity, and specificity were compared at the predefined thresholds and visualized using ROC curves, precision-recall curves, and forest plots, with the I^2^ statistic used to quantify heterogeneity across sites and vendors. Calibration, decision-curve, and reclassification analyses (net reclassification improvement [NRI] and integrated discrimination improvement [IDI]) were performed to assess clinical utility and the incremental improvement relative to PI-RADS, with multiple comparisons adjusted using Bonferroni correction to account for model dependence.

## 2 Patients and methods

### 2.1 Study population

Data for this study are from seven imaging centers participating in the Quantitative Prostate Imaging Consortium: University of California-San Diego (UCSD) Health; the Center for Translational Imaging and Precision Medicine (CTIPM) at UCSD; Massachusetts General Hospital (MGH); University of Rochester Medical Center (URMC); University of California-San Francisco (UCSF); University of Texas Health Sciences Center San Antonio (UTHSCSA); and University of Cambridge (Cambridge) [16]. The study was approved by the institutional review board (IRB) of UCSD. Each participating center collected and shared data after obtaining approval from the relevant local ethics committee. All research was performed in accordance with relevant regulations and guidelines, including the Declaration of Helsinki. At UCSD Health, UTHSCSA, and Cambridge, the data were collected prospectively as part of related projects. At the other centers, the data were collected retrospectively. Participants at UTHSCSA and Cambridge provided written informed consent, while a waiver of consent was approved by the relevant IRBs for the other centers for secondary use of routine clinical data.

While radiologists assigned PI-RADS scores at the participating centers, they had access to the imaging and whatever history was provided in the examination orders and/or clinical records at the time. All readers were board-certified, fellowship-trained radiologists with clinical privileges at their corresponding centers. Reads were according to PI-RADS v2.1 [5]. Neither RSIrs nor AI was available to radiologists at the time of their clinical reads. For final evaluation of performance in this study, patients with a prior biopsy were excluded, meaning that all radiologists were unaware of biopsy results at the time of the clinical read.

**Fig. 1:**
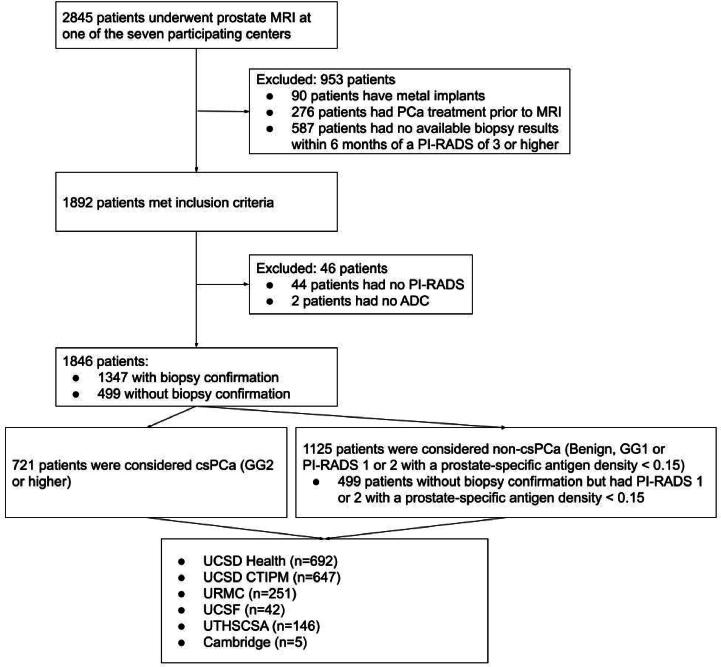
Flowchart of patient inclusion and exclusion. MRI = magnetic resonance imaging; PCa = prostate cancer. PI-RADS = Prostate Imaging-Reporting and Data System; csPCa = clinically significant PCa; ADC = apparent diffusion coefficient; GG = Gleason grade group; non-csPCa = non–clinically significant PCa; PSAD = prostate-specific antigen density (in ng/ml/cm^3^); UCSD = University of California-San Diego; CTIPM = Center for Translational Imaging and Precision Medicine; MGH = Massachusetts General Hospital; URMC = University of Rochester Medical Center; UCSF = University of California-San Francisco; UTHSCSA = University of Texas Health Sciences Center San Antonio.

Individuals were included if they were aged ≥18 years and underwent prostate MRI for suspected PCa or during active surveillance between January 2016 and March 2024. Patients who had prior treatment for PCa or had no biopsy result from within 6 mo of a positive MRI scan (PI-RADS ≥3) available were excluded. Patients with metallic implants were also excluded to avoid metal-induced imaging artifacts. The diagnosis of csPCa was confirmed on biopsy histopathology according to the standard practice at each center. These data have previously been analyzed in three studies to investigate the performance of RSIrs_max_ [14], [16], [31] (Fig. 1). One of these was a preliminary, single-center study in 151 patients [14]. The other two described evaluation of RSI in the same data set as in the present study [16], [31]. None of the analyses included any DL AI.

The patient characteristics by center are listed in Table 1.

**Table 1:** Patient characteristics by imaging center

Characteristic	UCSD Health	UCSD CTIPM	MGH	URMC	UCSF	UTHSCSA	Cambridge	Total
Cases (*n*)	692	647	63	251	42	146	5	1846
Median age, yr (IQR)	69 (64–75)	69 (64–74)	70 (64–76)	70 (64–74)	68 (64–72)	71 (66–76)	69 (69–70)	70 (64–75)
Median PSA, ng/ml (IQR)	7.2 (4.8–9.0)	7.8 (5.1–9.0)	7.1 (4.8–9.0)	6.4 (5.1–9.6)	9.0 (9.0–9.0)	8.4 (5.2–9.0)	3.6 (3.4–7.5)	7.4 (5.0–9.0)
csPCa, *n* (%)	252 (36)	269 (42)	27 (43)	107 (43)	25 (60)	40 (38)	1 (20)	721 (39)
Scanner manufacturer/model, *n* (%)								
GE Healthcare/Discovery MR750	546 (79)	470 (73)	0 (0)	0 (0)	0 (0)	0 (0)	5 (100)	1021 (55)
GE Healthcare/Signa Premier	146 (21)	177 (27)	63 (100)	0 (0)	42 (100)	0 (0)	0 (0)	428 (23)
Siemens Healthcare/Magnetom Skyra	0 (0)	0 (0)	0 (0)	251 (100.0)	0 (0)	58 (40)	0 (0)	309 (17)
Siemens Healthcare/Magnetom Trio	0 (0)	0 (0)	0 (0)	0 (0)	0 (0)	88 (60)	0 (0)	88 (4.8)
PI-RADS score, *n* (%)								
1	301 (44)	221 (34)	18 (29)	40 (16)	7 (17)	48 (33)	0 (0)	635 (35)
2	21 (3.0)	24 (3.7)	0 (0)	5 (2.0)	0 (0)	3 (2.1)	0 (0)	53 (2.9)
3	57 (8.2)	65 (10)	11 (18)	92 (37)	3 (7.1)	35 (24)	0 (0)	263 (14)
4	166 (24)	155 (24)	20 (32)	53 (21)	16 (38)	39 (27)	4 (80)	453 (25)
5	147 (21)	182 (28)	14 (22)	61 (24)	16 (38)	21 (14)	1 (20)	442 (24)
Biopsy status at MRI, *n* (%)								
BN-BC	309 (45)	259 (40)	38 (60)	233 (93)	20 (48)	17 (12)	0 (0)	876 (48)
PBI-NC	383 (55)	388 (60)	25 (40)	18 (7.2)	22 (52)	129 (88)	5 (100)	970 (53)
Gleason grade group, *n* (%)								
Benign	84 (12)	101 (16)	15 (24)	85 (34)	7 (17)	39 (27)	0 (0)	331 (18)
1	85 (12)	83 (13)	18 (29)	44 (18)	10 (24)	51 (35)	4 (80)	295 (16)
2	119 (17)	118 (18)	14 (22)	71 (28)	9 (21)	22 (15)	1 (20)	354 (19)
3	74 (11)	79 (12)	6 (9.5)	25 (10)	10 (24)	6 (4.1)	0 (0)	200 (11)
4	26 (3.8)	29 (4.5)	4 (6.3)	4 (1.6)	4 (9.5)	5 (3.4)	0 (0)	72 (3.9)
5	33 (4.8)	43 (6.6)	3 (4.8)	7 (2.8)	2 (4.8)	7 (4.8)	0 (0)	95 (5.1)
Biopsy pathology, *n* (%)								
Systematic Bx only	126 (18)	129 (20)	15 (24)	8 (3.2)	2 (4.8)	42 (29)	0 (0)	322 (17)
Targeted Bx only	1 (0.10)	15 (2.3)	20 (32)	103 (41)	28 (67)	5 (3.4)	5 (100)	177 (9.6)
Systematic Bx and targeted Bx	261 (38)	269 (42)	8 (13)	75 (30)	5 (12)	83 (57)	0 (0)	701 (38)
No Bx within 6 mo of MRI scan	305 (44)	234 (36)	20 (32)	65 (26)	7 (17)	16 (11)	0 (0)	646 (35)
Prostatectomy pathology available, *n* (%)	114 (17)	129 (20)	18 (29)	44 (18)	1 (2.4)	11 (7.5)	0 (0)	317 (17)

UCSD = University of California-San Diego; CTIPM = Center for Translational Imaging and Precision Medicine. MGH = Massachusetts General Hospital. URMC = University of Rochester Medical Center. UCSF = University of California-San Francisco. UTHSCSA = University of Texas Health Sciences Center San Antonio; IQR = interquartile range; PSA = prostate-specific antigen; csPCa = clinically significant prostate cancer (Gleason grade group ≥2); PI-RADS = Prostate Imaging-Reporting and Data System; MRI = magnetic resonance imaging; BN-BC = patients who were biopsy-naïve at the time of MRI and subsequently underwent biopsy; PBI-NC = patients with prior biopsy information at the time of MRI and no subsequent biopsy confirmation; Bx = biopsy.

### 2.2 Data acquisition and processing

RSI MRI acquisition required 2–3 min per patient. RSI data processing included correction for background noise, eddy currents, and gradient nonlinearities [32], [33], [34]. Correction for distortion caused by B_0_ inhomogeneity was applied to data acquired at UCSD CTIPM. ADC, DWI, and RSI data were resampled to the same image resolution as for the T2W data. Automated prostate contours were obtained using a US Food and Drug Administration–approved commercial product (OnQ Prostate, CorTechs.ai, San Diego, CA, USA).

For RSI data, the signal intensity for each b value was modeled as a linear combination of exponential decays representing four diffusion compartments (RSI-C_1_, RSI-C_2_, RSI-C_3_, and RSI-C_4_), each with a diffusion coefficient determined empirically in previous work [18]. The RSIrs biomarker is the intensity value of the RSI-C_1_ signal at a given voxel, normalized by the median T2W signal in the prostate. RSIrs_max_ is the maximum RSIrs value within a given patient’s prostate. Additional details regarding the RSI modeling are provided in Supplementary Table 1.

### 2.3 Models

**Fig. 2:**
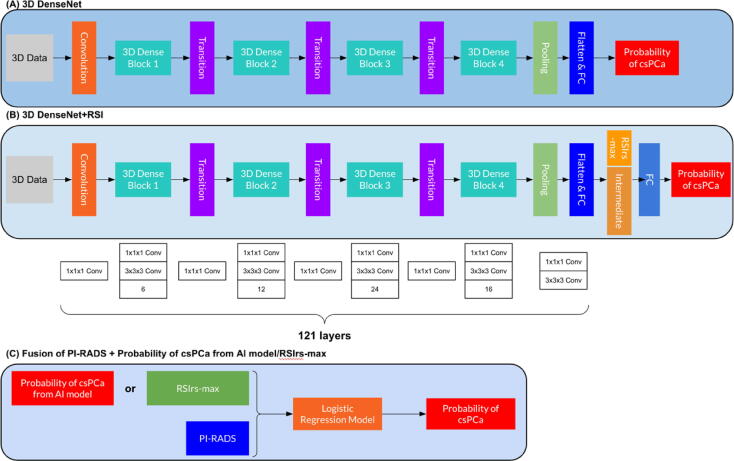
Overview of the experimental setup. (A) Three-dimensional (3D) data are used as input for 3D-DenseNet, followed by a convolution layer, several 3D dense blocks and transition layers. After the pooling layer and the flatten and fully connected (FC) layer, 3D-DenseNet returns a final csPCa classification. The input tensor size is three channels. (B) For 3D-DenseNet+RSI, the process before the flatten and FC layer is the same, followed by concatenation of the intermediate output from the flatten and FC layer with RSIrs_max_. After another FC layer, 3D-DenseNet+RSI returns the probability of csPCa as the final output. The input tensor size is five channels. For both models, the initial convolution layer expands the input from five to 64 channels. Dense block 1 (six layers, growth rate = 32) increases the channel count to 256, which is then reduced to 128 by transition 1. Dense block 2 (12 layers) increases the channels to 512, followed by a reduction to 256 via transition 2. Dense block 3 (24 layers) further expands the feature representation to 1024 channels before transition 3 reduces it to 512. Dense block 4 (16 layers) then produces the final 1024-channel feature map. For the 3D-DenseNet+RSI model, this 1024-channel tensor is reduced via adaptive average pooling and flattened into a 1024-dimensional vector, passed through a linear layer (1024 → 5), concatenated with the scalar RSIrs_max_ value, and finally mapped via a second linear layer (6 → out_channels, where out_channels = 2) to generate the final prediction. (C) PI-RADS and RSIrs_max_ or the csPCa output probabilities from the AI models are fused in a logistic regression model to produce the final csPCa probability. csPCa = clinically significant prostate cancer; PI-RADS = Prostate Imaging-Reporting and Data System; RSIrs_max_ = maximum restriction score from restriction spectrum imaging.

The performance of RSIrs_max_-only and PI-RADS-only models was previously analyzed for patient-level csPCa detection using univariable logistic regression [16]. Here, we compare the performance of DL models to those previously described logistic regression models. 3D-DenseNet [35] and 3D-DenseNet+RSI, both of which are three-dimensional (3D) densely connected convolutional networks, were trained to obtain the probability of csPCa with different modalities. Fig. 2 shows the details of the models and the modalities included.

The models were trained using binary cross-entropy loss with an AdamW optimizer, a learning rate of 0.001, and linear warm-up with a cosine annealing learning rate scheduler. More training details are described in the Supplementary material.

We also added either RSIrs_max_ or the output probabilities of the AI models to PI-RADS as inputs to a multivariable logistic regression model for comparison to either alone. The logistic regression coefficients, CIs, and calibration parameters are provided in Supplementary Table 2.

### 2.4 Implementation details

We used a leave-one-center-out (LOCO) approach to train and validate our models. Given that Cambridge had only five patients, we consistently included these data in the training split. The number of patients from each center is listed in Table 1. During each training iteration, data from one center were excluded for external validation, and an additional stratified 10% of the remaining training data (matched by csPCa status) was withheld for internal validation strictly for training control via best-epoch selection, rather than for model or hyperparameter selection, which resulted in six separate training rounds. There was no overlap between the training and validation cohorts for each training iteration to ensure complete data independence and prevent any information leakage. For validation, we applied the six trained models to their respective left-out data set. Finally, for model evaluation we focused on patients who were biopsy-naïve at the time of their MRI scan and then had biopsy confirmation after MRI acquisition (BN-BC cohort, *n* = 876).

The RSI-C_1_ and RSI-C_2_ components from RSI data, T2W, ADC and high-b-value DWI (high-b DWI) were included as the 3D data for input to the corresponding model. The Supplementary material describes preprocessing and augmentation for the input image data. Seven models were evaluated: model 1 = PI-RADS; model 2 = RSIrs_max_; model 3 (bpMRI) = T2W + ADC + high-b DWI; model 4 = bpMRI + RSI-C_1_,+ RSI-C_2_ + RSIrs_max_; model 5 = PI-RADS + RSIrs_max_; model 6 = output probabilities from model 3 + PI-RADS; and model 7 = output probabilities from model 4 + PI-RADS.

Two separate univariable logistic regression models with RSIrs_max_ or PI-RADS as input, and three multivariable logistic regression models with PI-RADS + RSIrs_max_, PI-RADS + output probability of model 3, and PI-RADS + output probability of model 4 as inputs were implemented using Scikit-learn [36].

All models, preprocessing, and data augmentation were implemented using the PyTorch toolbox and MONAI [37].

### 2.5 Statistical analysis

The precise location and extent of csPCa in each patient’s prostate are generally unknown, and targeted biopsy can have MRI-to-ultrasound registration or needle placement errors. Many cancers are also detected via systematic biopsy. We therefore focused on patient-level csPCa detection, which is a more reliable approach that addresses the key clinical question of whether to recommend an invasive biopsy. Under the LOCO framework, patient-level ROC curves and AUC values were computed by aggregating predicted probabilities obtained from the withheld center in each iteration. Specifically, performance metrics were calculated by restricting LOCO-generated predictions for each withheld center to the subset of patients who were biopsy-naïve at the time of MRI and subsequently underwent a biopsy (BN-BC, *n* = 876). This ensured that each patient was evaluated only once in an independent external validation setting. Performance metrics included AUC; specificity and the difference in specificity versus PI-RADS (ΔSpec) at fixed sensitivity of 0.90; NRI; and IDI. We made statistical comparisons via 10 000 bootstrapping samples to calculate 95% CIs using the percentile method (2.5th–97.5th percentiles of the bootstrap distribution), and *p* values for the difference in performance (AUC) between the models and PI-RADS [38] for the BN-BC cohort or per site-level clustering to demonstrate generalizability. A confusion matrix was generated for each model, as well as forest plots per site for AUC and for specificity at fixed sensitivity of 0.90, with heterogeneity quantified as I^2^. The analysis was for detection of GG ≥2 PCa (csPCa) versus GG 1 and benign cases. We applied a conservative Bonferroni correction to account for the seven comparisons (given that four of the seven models are combinations of the first three, these are not independent, and Bonferroni correction is even more conservative). As a result, statistical significance was determined using a two-sided test, with the significance threshold adjusted from α = 0.05 to α = 0.007.

We trained a model with bpMRI and compared its performance to separate univariable logistic regression models for PI-RADS alone and RSIrs_max_ alone. We also evaluated combining RSIrs_max_ and RSI with bpMRI to determine if RSIrs_max_ and RSI improve the performance over bpMRI alone.

Codes used in developing the DL model are available on GitHub (https://github.com/ESONG1999/Deep-learning-AI-and-RSI-for-patient-level-detection-of-csPCa-on-MRI).

## 3 Results

Data were acquired from seven imaging centers that used two scanner vendors, four scanner models, and 17 MRI scanners (Table 1 and Supplementary Tables 3 and 4). A total of 1892 patients met the inclusion criteria (Fig. 1).

### 3.1 Patient-level detection of csPCa

Table 2 shows performance results for the seven models for the BN-BC cases (*n* = 876).

**Table 2:** Performance results for models 1–7

Model a	Median result (95% confidence interval)					
	AUC	Threshold b	Specificity b	ΔSpecificity b	NRI	IDI
Model 1	0.75 (0.71–0.78)	PI-RADS 4 (3–4)	0.50 (0.14–0.56)	0 (0–0)	0 (0–0)	0 (0–0)
Model 2	0.73 (0.69–0.76)	202 (187–213)	0.32 (0.22–0.39)	−0.10 (−0.10 to −0.09)	0 (0–0)	−0.23 (−0.23 to −0.23)
Model 3	0.75 (0.72–0.78)	0.28 (0.21–0.33)	0.40 (0.30–0.48)	−0.01 (−0.02 to −0.01)	0.02 (0.02–0.02)	0 (0–0)
Model 4	0.77 (0.74–0.80)	0.23 (0.20–0.27)	0.39 (0.32–0.49)	−0.02 (−0.02 to −0.01)	0.06 (0.06–0.06)	0 (0–0)
Model 5	0.78 (0.75–0.81)	0.32 (0.31–0.35)	0.45 (0.38–0.52)	0.04 (0.04–0.04)	0.07 (0.07–0.07)	−0.01 (−0.01 to −0.01)
Model 6	0.78 (0.75–0.81)	0.30 (0.23–0.34)	0.45 (0.34–0.52)	0.03 (0.03–0.03)	0.09 (0.08–0.09)	0.03 (0.03–0.03)
Model 7	0.80 (0.77–0.83)	0.30 (0.25–0.35)	0.45 (0.35–0.53)	0.03 (0.03–0.04)	0.11 (0.11–0.11)	0.04 (0.04–0.04)

AUC = area under the receiver operating characteristic curve; PI-RADS = Prostate Imaging-Reporting and Data System; ΔSpecificity = difference in specificity in comparison to PI-RADS; NRI = net reclassification improvement; IDI = integrated discrimination improvement; RSIrs_max_ = maximum restriction score from restriction spectrum imaging; bpMRI = biparametric MRI; magnetic resonance imaging; RSI-C_1_/C_2_ = compartment 1/2 on RSI.

a Model 1: logistic regression model with PI-RADS input. Model 2: logistic regression model with RSIrs_max_ input. Model 3: 3D-DenseNet model with T2-weighted imaging, the apparent diffusion coefficient, and high-b diffusion-weighted imaging input (bpMRI). Model 4: 3D-DenseNet+RSI model with bpMRI, RSI-C_1_, RSI-C_2_, and RSIrs_max_ input. Model 5: logistic regression model with PI-RADS and RSIrs_max_ input. Model 6: logistic regression model with PI-RADS and the output probability of model 3. Model 7: logistic regression model with PI-RADS and the output probability of model 4.

b At fixed sensitivity of 0.90.

In comparison to PI-RADS alone (AUC 0.75, 95% CI 0.71–0.78), both RSIrs_max_ (AUC 0.73, 95% CI 0.69–0.76; *p* = 0.3) and model 3 (bpMRI DL AI model; AUC 0.75, 95% CI 0.72–0.78; *p* = 0.9) yielded similar performance. Addition of RSI and RSIrs_max_ to the DL bpMRI model did not result in a statistically significant improvement in csPCa detection (model 4; AUC 0.77, 95% CI 0.74–0.80; *p* = 0.2).

Addition of PI-RADS to RSIrs_max_ (AUC 0.78, 95% CI 0.75–0.81; *p* < 0.001) or to model 3 (AUC 0.78, 95% CI 0.75–0.81; *p* < 0.001) or model 4 (AUC 0.80, 95% CI 0.77–0.82; *p* < 0.001) yielded significantly better patient-level csPCa detection in comparison to PI-RADS alone.

It should be noted that discrimination metrics such as AUC primarily reflect the informativeness and variability of the predictors available in the data set rather than the modeling technique itself. Accordingly, the differences in AUC observed across models in this study should be interpreted in the context of the underlying input features and their distribution within the cohort, rather than as a direct comparison of algorithmic complexity or modeling approach.

The threshold at fixed sensitivity of 0.90 was PI-RADS 4 (95% CI 3–4) for the PI-RADS model, 202 (95% CI 187–213) for the RSIrs_max_ model, 0.28 (95% CI 0.21–0.33) for model 3, 0.23 (95% CI 0.20–0.27) for model 4, and a median of approximately 0.30 for all of the combination models (model 5–7). Here, the 95% CI for the PI-RADS threshold reflects the variability in PI-RADS cutoff selected across the 10 000 bootstrap resamples required to achieve 0.90 sensitivity, rather than statistical uncertainty for a continuous threshold estimate.

Results for median ΔSpec in comparison to PI-RADS at fixed sensitivity of 0.90 were all negative for RSIrs_max_ (−0.10, 95% CI −0.10 to −0.09), model 3 (−0.01, 95% CI −0.02 to −0.01), and model 4 (−0.02, 95% CI −0.02 to −0.01). Addition of PI-RADS to these models resulted in positive median ΔSpec values of 0.03–0.04.

For reclassification performance in comparison to PI-RADS, the lowest improvement was observed for RSIrs_max_, with NRI of 0 (95% CI 0–0) and IDI of −0.23 (95% CI −0.23 to −0.23). Models 3 and 4 showed small but consistent gains, with NRI values of 0.02 (95% CI 0.02–0.02) and 0.06 (95% CI 0.06–0.06), and IDI values of 0 (95% CI 0–0) and −0.01 (95% CI −0.01 to −0.01), respectively. By contrast, addition of imaging-based features to PI-RADS yielded substantially greater improvements, with NRI increasing to 0.07–0.11 (95% CI 0.07–0.11) and IDI to 0.03–0.04 (95% CI 0.03–0.04) across the three combination models. These results indicate that while single-modality models offer a limited benefit over PI-RADS, integration with PI-RADS produces consistent and meaningful gains in patient-level reclassification performance. Per-site metrics for the above analyses are provided in Supplementary Table 5.

**Fig. 3:**
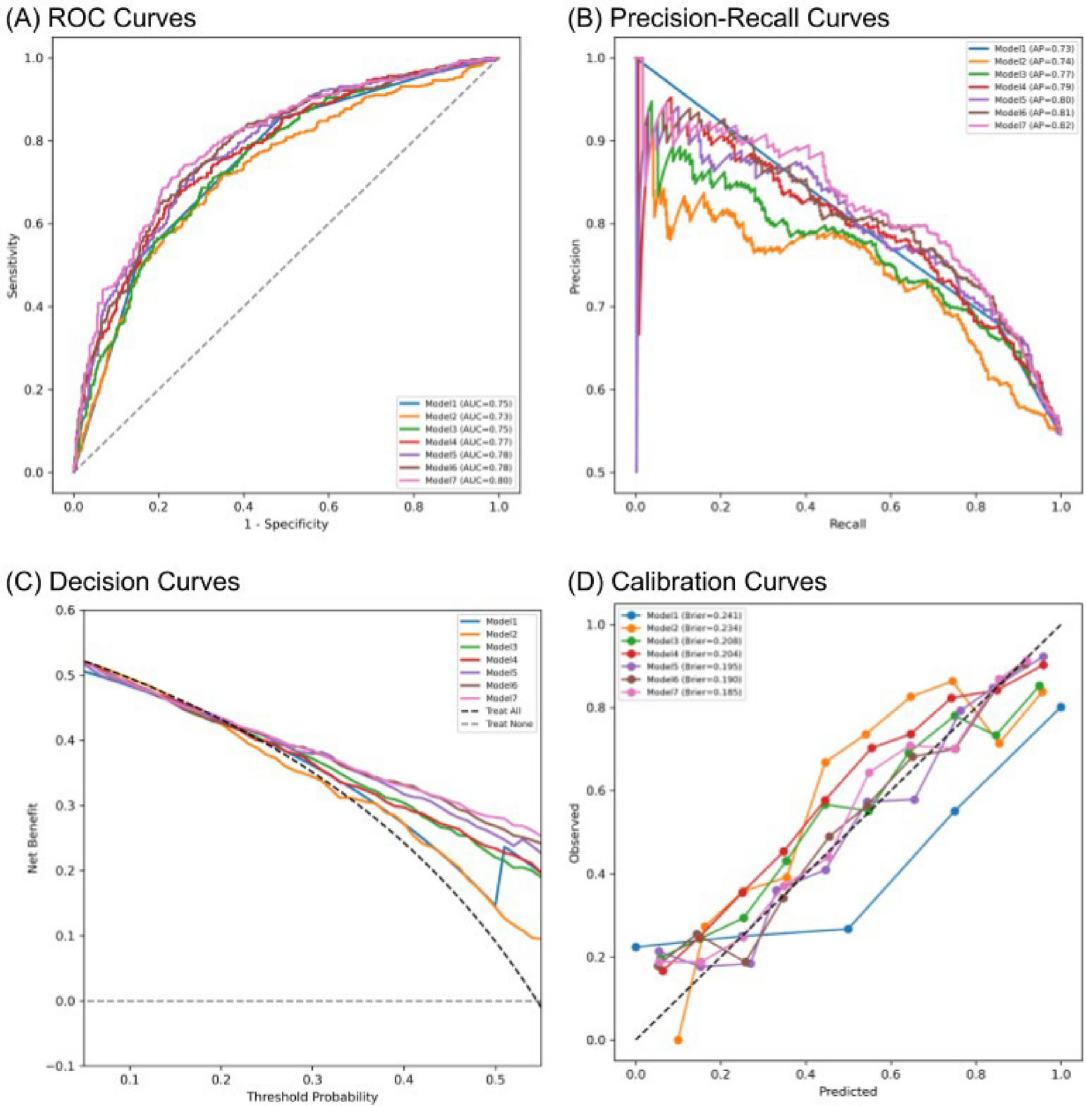
(A) Receiver operating characteristic curves. (B) Precision recall curves. (C) Decision curves. (D) Calibration curves, average precision. Model 1 is the logistic regression model with PI-RADS input. Model 2 is the logistic regression model with RSIrs_max_ input. Model 3 is 3D-DenseNet with T2-weighted imaging, the apparent diffusion coefficient, and high-b-value diffusion-weighted imaging as input (bpMRI). Model 4 is 3D-DenseNet+RSI with bpMRI, RSI-C_1_, RSI-C_2_, and RSIrs_max_ as input. Model 5 is the logistic regression model with PI-RADS and RSIrs_max_ as input. Model 6 is the logistic regression model with PI-RADS and the output probability of model 3. Model 7 is the logistic regression model with PI-RADS and the output probability of model 4. AP = average precision; AUC = area under the curve; bpMRI = biparametric magnetic resonance imaging; Brier = Brier score; PI-RADS = Prostate Imaging-Reporting and Data System; RSI-C_1_/C_2_ = compartment 1/2 on restriction spectrum imaging; RSIrs_max_ = maximum restriction score from restriction spectrum imaging.

Fig. 3 shows ROC, precision recall, decision, and calibration curves for the cohort of BN-BC cohort (*n* = 876). Results for the ROC curves in terms of AUC have already been described. The precision recall curves show that models 5–7 achieved the highest discrimination performance, with model 7 yielding the highest average precision (AP = 0.82), followed by model 6 (AP = 0.81) and model 5 (AP = 0.80). These advanced models retained higher precision across a broad range of recall values, whereas PI-RADS, RSIrs_max_, and models 3 and 4 showed notably lower performance (AP range 0.73–0.79), with PI-RADS performing the worst. Consistent with these findings, the decision curves show that models 5–7 provided a higher net benefit (NB) across the threshold probabilities evaluated, while NB was comparatively lower for PI-RADS, RSIrs_max_, and models 3 and 4, with substantial overlap of the curves. For the aggregated cohort, models 5–7 had lower Brier scores (model 7: 0.19; model 6: 0.19; model 5: 0.20) and calibration curves that were closer to the ideal 45° line in comparison to PI-RADS, RSIrs_max_, and models 3 and 4. However, site-level analysis (Supplementary Fig. 1) revealed substantial heterogeneity in calibration performance across institutions. While models 5–7 generally retained lower Brier scores relative to PI-RADS at most sites, marked departures from the ideal 45° line were observed for several centers, which indicated that the absolute probability estimates are sensitive to the local setting. This between-site variability probably reflects differences in underlying disease prevalence, patient case mix, referral patterns, and imaging protocols specific to each institution. Consequently, while the advanced models may offer better ranking of risk in comparison to standard methods, it should not be assumed that the absolute predicted probabilities are universally transferable. These findings underscore the fact that local recalibration is probably necessary to ensure accurate probability estimates before clinical implementation in new settings.

Supplementary Fig. 2 shows the confusion matrix for each model. Forest plots for AUC (Supplementary Fig. 3) and for specificity at fixed sensitivity of 0.90 (Supplementary Fig. 4) present results by site. According to the forest plots for AUC, RSIrs_max_ (I^2^ = 22%) and model 4 (I^2^ = 31%) showed low heterogeneity and the other models showed no heterogeneity (I^2^ = 0%) across sites. Forest plots for specificity at fixed sensitivity of 0.90 revealed that PI-RADS (I^2^ = 76%) showed high heterogeneity, model 4 (I^2^ = 52%) showed moderate heterogeneity, and the other models showed no heterogeneity (I^2^ = 0%) across sites. I^2^ is reported for exploratory purposes; with few sites and imbalanced samples, heterogeneity estimates may be unstable.

Occlusion sensitivity maps for model 4 generated for the interpretability of the DL model are shown in Supplementary Fig. 5.

## 4 Discussion

Previous work demonstrated the promising utility of RSIrs_max_ as a quantitative imaging biomarker for csPCa [14], [15], [16], [18], [19], [39]. Here we explored DL AI as an alternative or complementary approach for reproducible, objective interpretation of prostate MRI. We observed comparable performance for csPCa detection using PI-RADS, a bpMRI AI model, and RSIrs_max_. While training of AI models using bpMRI and RSI data (RSIrs_max_ alone or with full RSI volumes) resulted in a numerical improvement in detection, the difference was not statistically significant in comparison to PI-RADS. However, addition of PI-RADS to any of these three models significantly enhanced the detection performance in comparison to PI-RADS alone. ΔSpec at fixed sensitivity of 0.90 for the combination models ranged from 0.03 to 0.04, which would correspond to an expected reduction of approximately two or three unnecessary biopsies per 100 men given the csPCa prevalence of 39% reported in Table 1. Decision curve analysis (Fig. 3C) showed that the combination models provided higher NB than PI-RADS and single-modality approaches, with the advantage most pronounced at moderate to high threshold probabilities. This suggests that the combination models offer better clinical utility in comparison to default strategies, conditional on the use of a higher decision threshold rather than a uniform benefit across the entire probability spectrum. RSIrs_max_ availability remains limited at present; however, RSI requires only 2–3 min to acquire and provides rapid, quantitative, vendor-independent, and standardized data to support radiologist interpretation, and could augment PI-RADS scoring.

An advantage of a bpMRI AI tool over RSIrs_max_ is that it may be applicable to data sets lacking DWI compatible with calculation of RSIrs_max_. The DL model does not rely on radiologist expertise and offers results comparable to PI-RADS, which could make it a useful tool in helping less experienced prostate radiologists achieve more accurate performance to address the growing shortage of subspecialist expert radiologists [6].

Addition of RSIrs_max_ or a DL model to PI-RADS yielded statistically significantly better results than PI-RADS alone. The most significant problem with PI-RADS is its considerable variability, which is a problem exacerbated by the learning curve and the need for expert radiologists who subspecialize in prostate MRI. This problem is well documented in the literature [7], [40]. Tools such as RSIrs and AI models with comparable performance to expert radiologists (as shown in the present paper) could be an additional safety check for all reads and could potentially improve the performance of radiologists, especially those inexperienced in prostate MRI. We are evaluating this hypothesis in an ongoing multicenter trial (ART-Pro; NCT06579417) [40].

The performance results for our models are concordant with recent studies on AI for prostate MRI that also showed patient-level csPCa detection comparable to PI-RADS [26], [41]. For example, in the PI-CAI challenge, an AI model was developed as a single combination of the five best-performing models (among 293 submitted for the challenge) and performed similarly to radiologists [26]. Of note, beyond imaging data, the PI-CAI model also included age, prostate-specific antigen (PSA) level, prostate volume, and scanner model. The inclusion of these clinical and technical variables, together with a large training data set (10 207 MRI examinations from 9129 patients), contributed to statistically significant better performance than the pooled performance of radiologists who participated in a retrospective reader study (but not better than real-time clinical PI-RADS interpretation of the same scans). Another recent study described two models developed for patient-level detection: one with only imaging data, and another that combined imaging with PSA and PSA density [41]. The performance of the imaging-only model—which used ADC, T2W, high-b DWI (*b* = 1600), and dynamic contrast-enhanced MRI as inputs and was trained on data from 5035 examinations—was comparable to that of human experts on the external testing set. When combined with the radiologists’ PI-RADS scores, the model also achieved a statistically significant improvement in performance, consistent with our findings.

The quality of imaging and of interpretation is important for a diagnostic imaging program. Real-world clinical data are inherently subject to the challenges of real-time clinical workflows and variation across centers, scanners, scanner operators, and radiologist readers. It is not possible to simply compare AUC values across different studies because AUC depends on case mix, disease prevalence, reference standards, imaging protocols, scanners (hardware and software), and model inputs (eg, imaging only vs imaging and clinical variables). All centers in our study are high-volume academic medical centers where clinical interpretation was performed by board-certified, fellowship-trained abdominal/genitourinary radiologists, which partly mitigates concerns regarding image quality. Radiologists contributing the clinical reads typically had several years to decades of postfellowship experience; thousands of prostate MRI examinations interpreted (thus meeting the European Society of Urogenital Radiology/EAU Section of Urological Imaging criterion for “expert” level [42]); and routine involvement in multidisciplinary PCa care, including biopsy correlation and clinical follow-up. All contributing centers have robust local quality standards and review processes. Internal reviews demonstrated that ≥95% of clinical prostate MRI examinations meet diagnostic quality standards, consistent with Prostate Imaging-Quality v1 scores ≥3 (and most ≥4) [43].

Limitations of our study include those typical for prostate MRI. In particular, biopsy is an imperfect gold standard owing to possibility of missing csPCa, even when using MRI for targeting. This represents a fundamental challenge in the training of AI models and in evaluating the performance of clinical prostate MRI. Large studies and meta-analyses have confirmed overall good performance of clinical interpretation despite this uncertainty. Individuals with hip implants were excluded because of known potential to cause severe artifacts on MRI; quantitative RSIrs may require calibration and additional attention to artifacts before application to patients with hip implants [44]. In addition, our data set included both prospective and retrospective cohorts, which may differ in acquisition consistency and patient selection processes. Our LOCO evaluation mitigates site-specific bias, but residual design-related differences might still influence the performance observed and limit the generalizability of the results. Our data set does not include case-level information about the individual radiologists, although all clinical reads were performed by experienced subspecialists. There was no central reading, so inter-reader variability could not be assessed. We did not explore potential nonlinear relationships between PI-RADS and csPCa. While calibration was assessed, further prospective recalibration and external validation are needed before clinical use. To the best of our knowledge, this is the largest study to date combining AI and an advanced MRI biomarker for PCa. Because RSI is not yet widely available, the current applicability of RSIrs_max_ and RSI-based models is limited. An ongoing prospective trial is evaluating whether incorporation of these tools into clinical workflows improves biopsy decision-making, reduces unnecessary procedures, and enhances csPCa detection in routine practice.

## 5 Conclusions

We found that addition of either RSIrs_max_ or an AI model to PI-RADS slightly outperformed PI-RADS alone, which suggests that both could serve as valuable complements to human expertise. Prospective studies should evaluate whether meaningful performance gains can be achieved by incorporating these tools into clinical workflows for radiologists lacking specific expertise in prostate MRI.

  ***Author contributions*:** Tyler M. Seibert had full access to all the data in the study and takes responsibility for the integrity of the data and the accuracy of the data analysis.

  *Study concept and design:* Song, Seibert, Rojo Domingo, Conlin, Do.

*Acquisition of data:* All authors.

*Analysis and interpretation of data:* Song, Seibert, Rojo Domingo, Conlin, Do.

*Drafting of the manuscript:* Song, Seibert.

*Critical revision of the manuscript for important intellectual content:* All authors.

*Statistical analysis:* Seibert, Dale.

*Obtaining funding:* Seibert.

*Administrative, technical, or material support:* Seibert.

*Supervision:* Seibert.

*Other:* None.

  ***Financial disclosures*:** Tyler M. Seibert certifies that all conflicts of interest, including specific financial interests and relationships and affiliations relevant to the subject matter or materials discussed in the manuscript (eg, employment/affiliation, grants or funding, consultancies, honoraria, stock ownership or options, expert testimony, royalties, or patents filed, received, or pending), are the following: Daniel J.A. Margolis reports a clinical advisor role for Stratagen Bio and an ad hoc consultant role for Guerbet and Promaxo. Sean A. Woolen reports funding from an ARRS Scholarship for Professional Development and institutional investigator-initiated research grants from Siemens. Sophia Kamran reports spouse employment with Sanofi. Anders M. Dale reports a founder role for, equity interest in, and a scientific advisory board role for CorTechs Labs; a scientific advisory board role for Healthlytix; and research funding from GE Healthcare. Rebecca Rakow-Penner reports consultant roles for Human Longevity Inc, Curemetrix, and Bayer; stock options in CorTechs Labs and Curemetrix; an advisory board role for Imagine Scientific; and a research agreement with GE Healthcare. Michael A. Liss reports a founder/president role for Oncobiomix outside the scope of this work. Tyler M. Seibert reports honoraria from Multimodal Imaging Services Corporation, Varian Medical Systems, Janssen, and WebMD; and equity interest in and a scientific advisory board role for CorTechs Labs. These companies might potentially benefit from the research results. The terms of the above arrangements have been reviewed and approved by the University of California-San Diego in accordance with its conflict-of -interest policies. The remaining authors have nothing to disclose. A product from CorTechs Labs (OnQ Prostate) was used for automated segmentation of the prostate. Authors with control of the study data include Yuze Song, Mariluz Rojo Domingo, and Tyler M. Seibert; none of these authors has a fiduciary responsibility to CorTechs Labs. The remaining authors have nothing to disclose.

  ***Funding/Support and role of the sponsor:*** This study received funding from the 10.13039/100000002National Institutes of Health (grants NIH/NIBIB K08EB026503 and NIHUL1TR000100), the American Society for Radiation Oncology, the 10.13039/100000892Prostate Cancer Foundation (grant PCF20YOUN01), and the 10.13039/100010210US Department of Defense (grant DOD/CDMRPPC220278). The funding bodies played no direct role in the study.
