# Supplementary material for: Deep Learning Artificial Intelligence and Restriction Spectrum Imaging for Patient-level Detection of Clinically Significant Prostate Cancer on Biparametric Magnetic Resonance Imaging
Source: Eur Urol Open Sci. 2026 Feb 6;85:49–59. doi: 10.1016/j.euros.2026.01.014 (PMC12905774; doi:10.1016/j.euros.2026.01.014)
Supplement: Supplementary Data 1 [file mmc1.docx]

**Supplementary Materials – Figure**

**
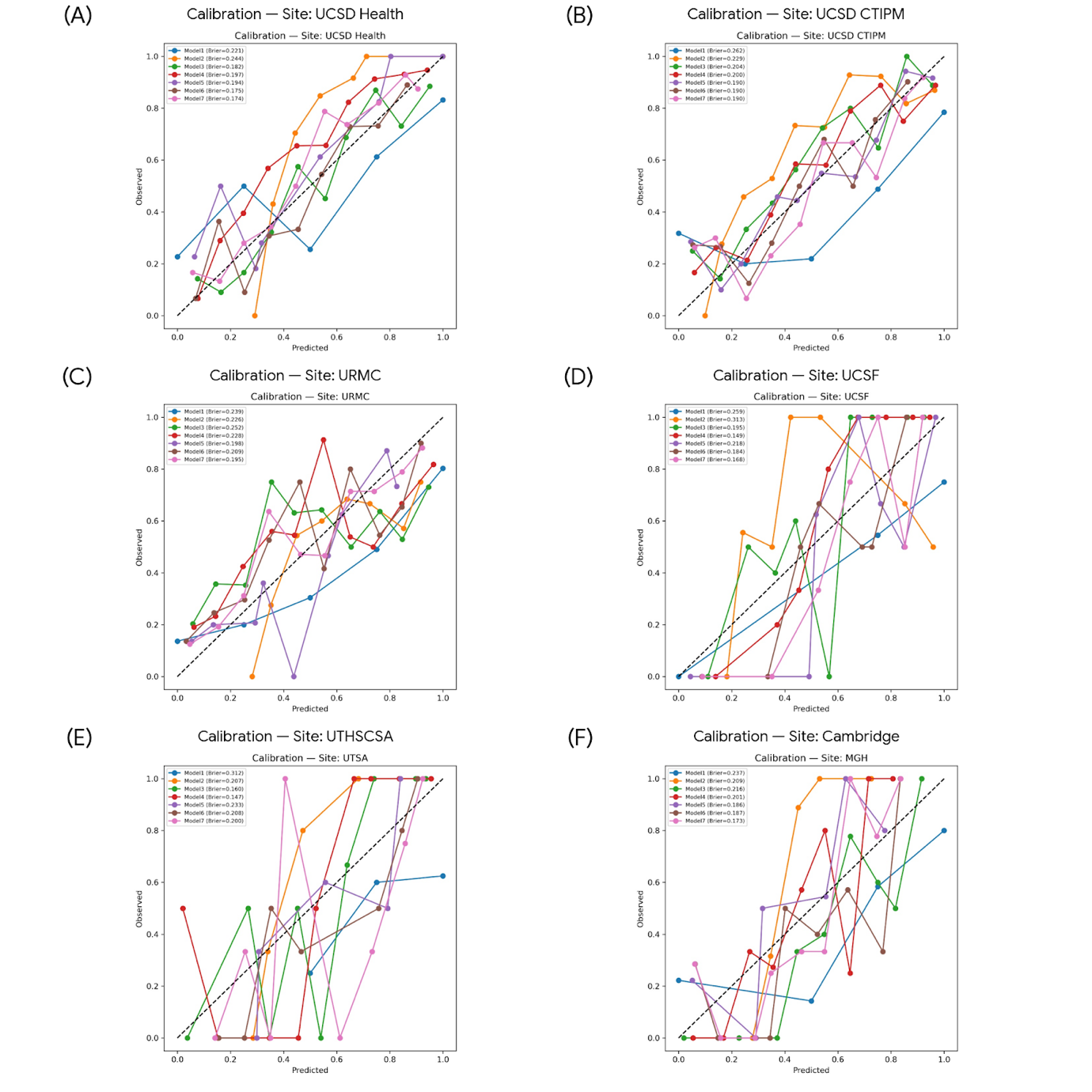
**

Supplementary Figure 1. Calibration curves across sites, Brier refers to Brier Score. Model 1 refers to the logistic regression model with PI-RADS input; Model 2 refers to the logistic regression model with RSIrs-max input; Model 3 refers to the 3D DenseNet with T2w, ADC and high-b DWI input (bpMRI); Model 4 refers to the 3D-DenseNet+RSI with bpMRI, RSI-C1, RSI-C2 (RSI) and RSIrs-max input; Model 5 refers to the logistic regression model with PI-RADS and RSIrs-max input; Model 6 refers to the logistic regression model with PI-RADS and the output probability of Model 3; Model 7 refers to the logistic regression model with PI-RADS and the output probability of Model 4.


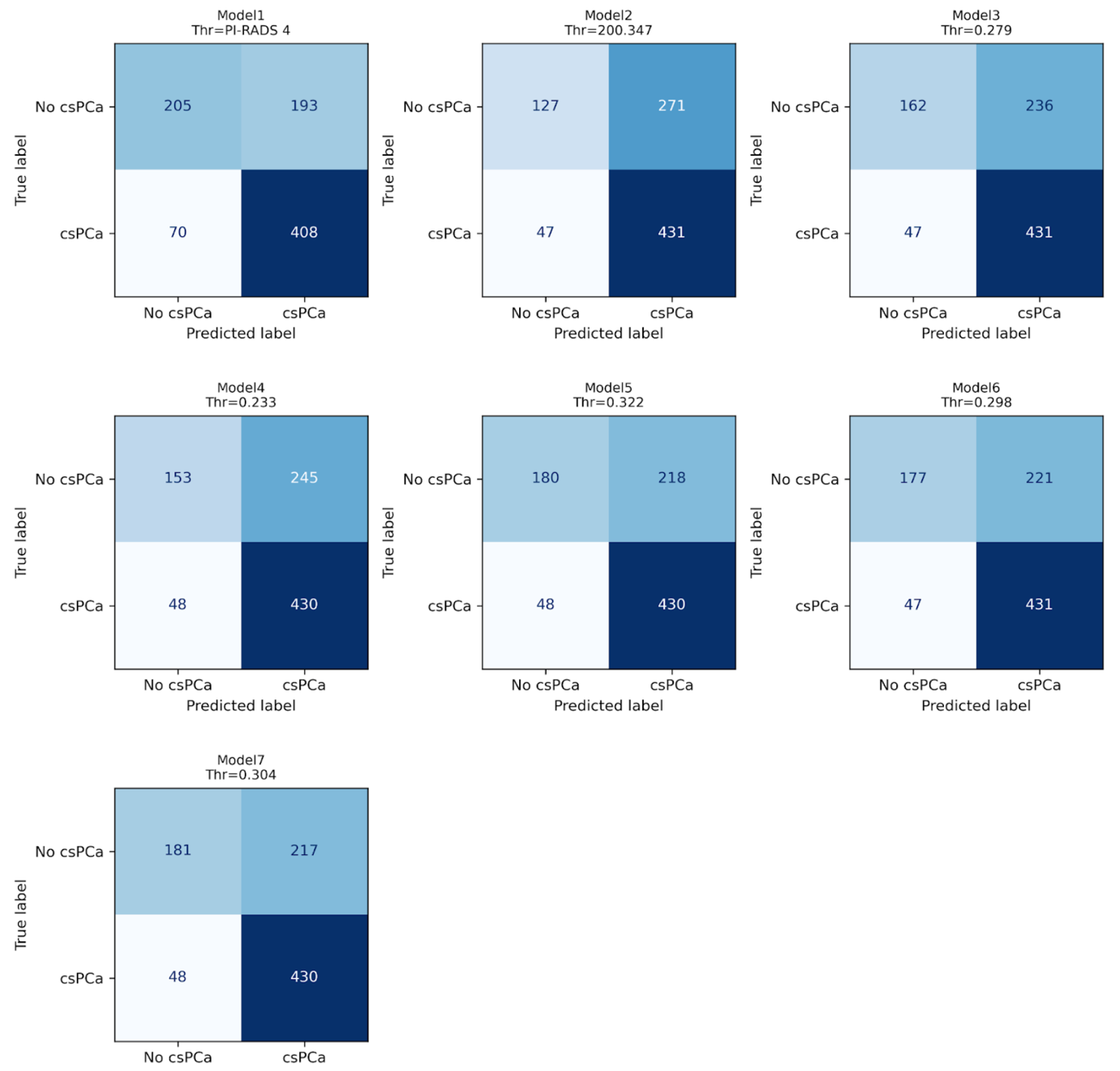


Supplementary Figure 2. Confusion Matrix for different models at fixed sensitivity of 0.90. Thr is the threshold for different model at fixed sensitivity of 0.90. Model **1** refers to the logistic regression model with PI-RADS input; Model **2** refers to the logistic regression model with RSIrs-max input; Model **3** refers to the 3D DenseNet with T2w, ADC and high-b DWI input (bpMRI); Model **4** refers to the 3D-DenseNet+RSI with bpMRI, RSI-C_1_, RSI-C_2_ (RSI) and RSIrs-max input; Model **5** refers to the logistic regression model with PI-RADS and RSIrs-max input; Model **6** refers to the logistic regression model with PI-RADS and the output probability of Model **3**; Model **7** refers to the logistic regression model with PI-RADS and the output probability of Model **4**.


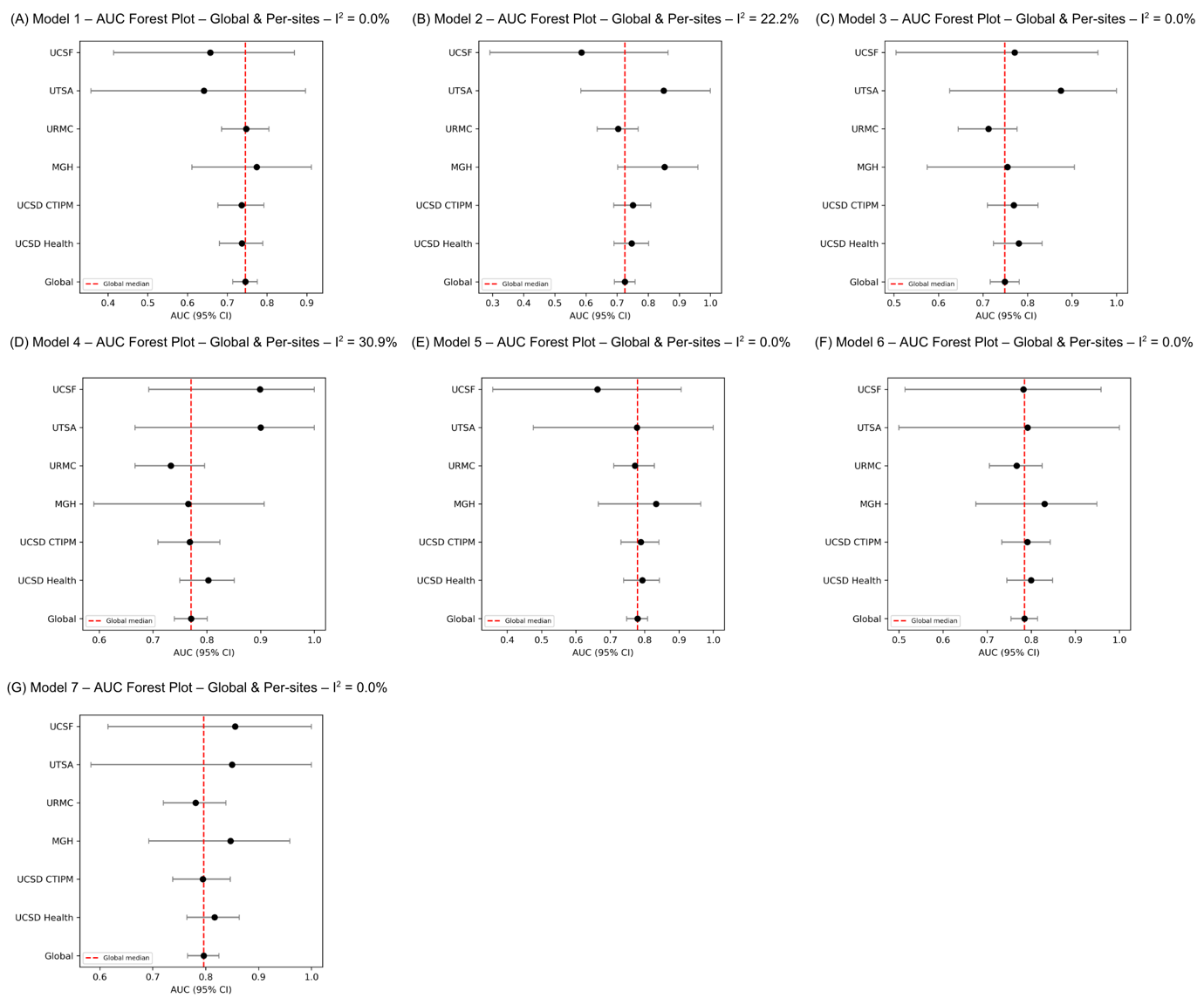


Supplementary Figure 3. AUC Forest plots for different models. I^2^ is the heterogeneity for different models for all patients (Global) and per-sites. Model **1** refers to the logistic regression model with PI-RADS input; Model **2** refers to the logistic regression model with RSIrs-max input; Model **3** refers to the 3D DenseNet with T2w, ADC and high-b DWI input (bpMRI); Model **4** refers to the 3D-DenseNet+RSI with bpMRI, RSI-C_1_, RSI-C_2_ (RSI) and RSIrs-max input; Model **5** refers to the logistic regression model with PI-RADS and RSIrs-max input; Model **6** refers to the logistic regression model with PI-RADS and the output probability of Model **3**; Model **7** refers to the logistic regression model with PI-RADS and the output probability of Model **4**.


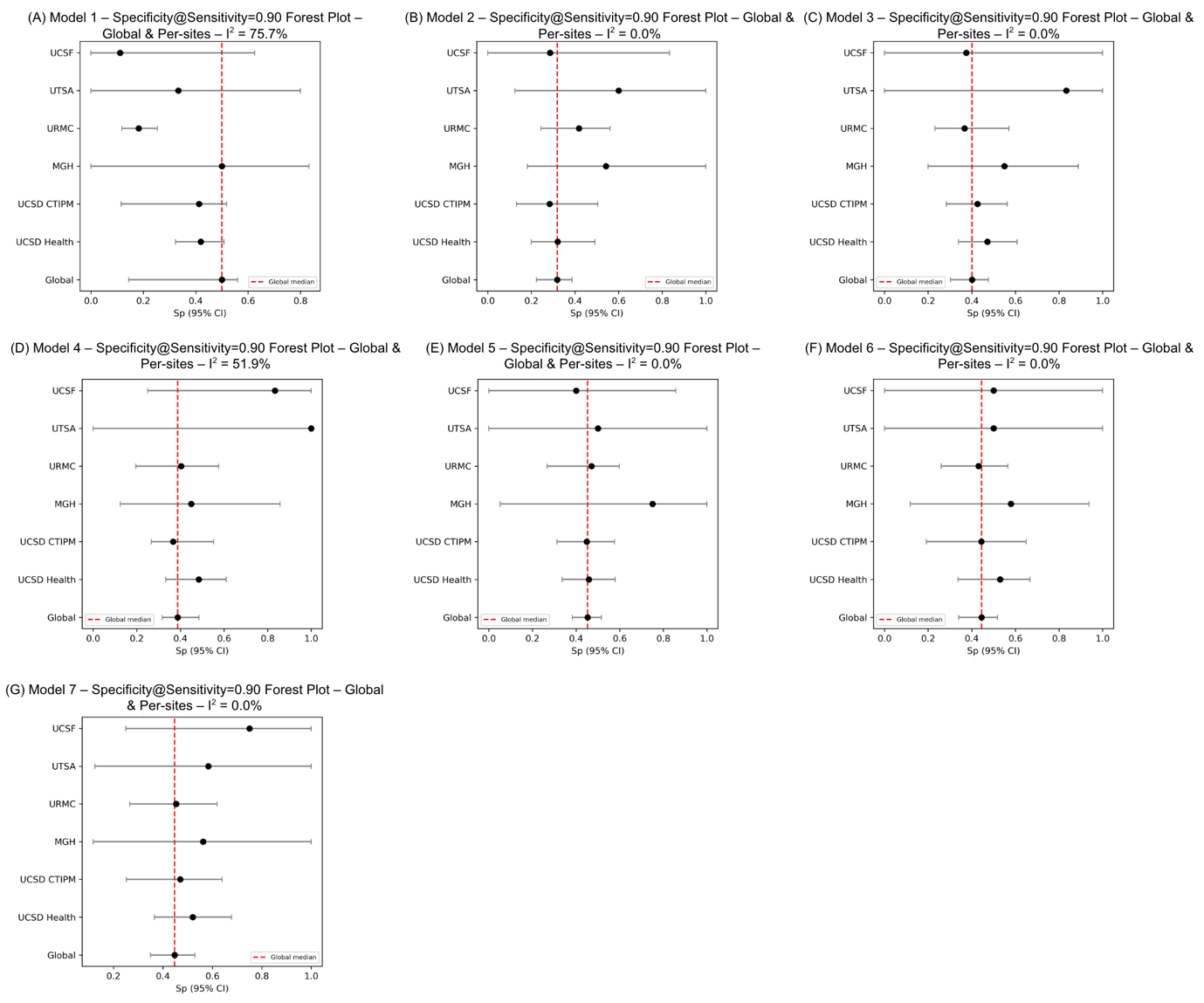


Supplementary Figure 4. Specificity at fixed sensitivity of 0.90 Forest plots for different models. I^2^ is the heterogeneity for different models for all patients (Global) and per-sites. Model **1** refers to the logistic regression model with PI-RADS input; Model **2** refers to the logistic regression model with RSIrs-max input; Model **3** refers to the 3D DenseNet with T2w, ADC and high-b DWI input (bpMRI); Model **4** refers to the 3D-DenseNet+RSI with bpMRI, RSI-C_1_, RSI-C_2_ (RSI) and RSIrs-max input; Model **5** refers to the logistic regression model with PI-RADS and RSIrs-max input; Model **6** refers to the logistic regression model with PI-RADS and the output probability of Model **3**; Model **7** refers to the logistic regression model with PI-RADS and the output probability of Model **4**.


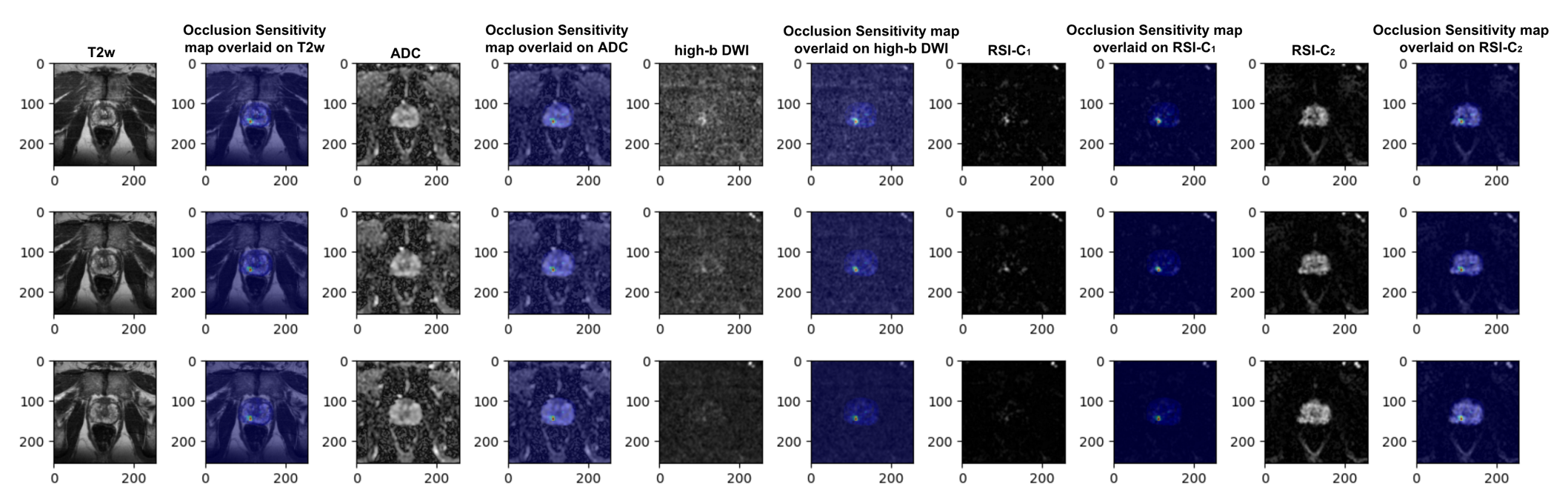


Supplementary Figure 5. Occlusion Sensitivity Maps of Model 4 Overlaid on MRI images from a 57-year-old male with pathology confirmed Gleason Score 4 + 3 (GG 3) cancer at the right and left posterior near the apex of the prostate. T2w, ADC, high-b DWI, RSI-C1 and RSI-C2 images from left to right with occlusion sensitivity maps from Model 4 overlaid at the right of each MRI images. The radiologists graded this examination as PI-RADS 4 for the lesion at the right peripheral zone at the posterior medial prostate with apex.
