# Supplementary material for: Deep Learning Artificial Intelligence and Restriction Spectrum Imaging for Patient-level Detection of Clinically Significant Prostate Cancer on Biparametric Magnetic Resonance Imaging
Source: Eur Urol Open Sci. 2026 Feb 6;85:49–59. doi: 10.1016/j.euros.2026.01.014 (PMC12905774; doi:10.1016/j.euros.2026.01.014)
Supplement: Supplementary Data 2 [file mmc2.docx]

**Supplementary Materials – Info & Tables**

**RSI model equation**

The RSI model equation shows the relationship between the DWI signal from the four compartments and the DWI signal intensity at a specific *b*-value. $S\left( b \right)$ represents the measured DWI signal intensity at a specific *b*-value.

$S\left( b \right) = \sum_{i = 1}^{4} C_{i}e^{-bD_{i}}$ (1)

The signal is computed through the combination of exponential decays, each representing one of four diffusion compartments. $C_{i}$ is the signal contribution of a particular compartment to the overall signal and is determined through model-fitting. $D_{i}$ is the diffusion coefficient that is empirically determined for each of the four compartments $\left( C_{i} \right)$. The detailed parameter for each RSI compartments is shown in Supplementary table 1.

**Preprocessing and augmentation of Images**

All 3D-data was first resampled onto the voxel space of the corresponding T2w volume. Then, the resolution of all data was resampled to 0.5 x 0.5 x 3 mm^3^. The 3D data were cropped to 256 x 256 x 32 voxels and normalized to have a zero mean and unit standard deviation. Data augmentation techniques included: random axis mirror flip with a probability of 0.2 for all three dimensions, random intensity scaling in the range (0.9, 1.1) and random intensity shift in the range (-0.1, 0.1).

**Model Training**

The models were trained on 1 NVIDIA GeForce RTX 2080 Ti GPU, with a batch size of 8 for a total of 120 epochs. The learning rate for both models was set to 0.001. A linear warm-up with a cosine annealing learning rate scheduler was used with 60 warm up epochs. For the training of each leave-one-center out validation, we picked the model that achieved the best accuracy at a probability threshold of 0.5 on the 10% withholding part of the training data.

| **RSI compartment** | **Fixed Diffusion Coefficient (s/mm^2^)** |
| --- | --- |
| Restricted Diffusion $\left( C_{1} \right)$ | $1.1 \times{10}^{-4} \left( D_{1} \right)$ |
| Hindered Diffusion $\left( C_{2} \right)$ | $1.8 \times{10}^{-3} \left( D_{2} \right)$ |
| Free Diffusion $\left( C_{3} \right)$ | $3.6 \times{10}^{-3} \left( D_{3} \right)$ |
| Vascular Flow $\left( C_{4} \right)$ | $0.1220 \left( D_{4} \right)$ |

Supplementary Table 1. RSI compartment and its fixed diffusion coefficient.

| **Dataset** | **Model** | **Calibration Intercept** | **Calibration Slope** | **Variable** | **Coefficient** | **95% CI Lower** | **95% CI Upper** |
| --- | --- | --- | --- | --- | --- | --- | --- |
| UCSD Health | Model 5 | -1.3 | 1.7 | const | -3.8 | -4.3 | -3.3 |
|  |  |  |  | PI-RADS | 0.93 | 0.81 | 1.1 |
|  |  |  |  | RSIrs-max | 0 | 0 | 0 |
| UCSD Health | Model 6 | -1.4 | 1.9 | const | -4.2 | -4.7 | -3.7 |
|  |  |  |  | PI-RADS | 0.81 | 0.69 | 0.93 |
|  |  |  |  | Output of Model 3 | 2.4 | 1.8 | 3.1 |
| UCSD Health | Model 7 | -1.4 | 2.0 | const | -4.3 | -4.8 | -3.8 |
|  |  |  |  | PI-RADS | 0.78 | 0.66 | 0.90 |
|  |  |  |  | Output of Model 4 | 2.9 | 2.2 | 3.6 |
| UCSD CTIPM | Model 5 | -1.7 | 2.3 | const | -4.8 | -5.4 | -4.3 |
|  |  |  |  | PI-RADS | 0.94 | 0.81 | 1.1 |
|  |  |  |  | RSIrs-max | 0 | 0 | 0.01 |
| UCSD CTIPM | Model 6 | -1.8 | 2.4 | const | -4.6 | -5.2 | -4.1 |
|  |  |  |  | PI-RADS | 0.89 | 0.77 | 1.0 |
|  |  |  |  | Output of Model 3 | 2.5 | 1.8 | 3.1 |
| UCSD CTIPM | Model 7 | -1.8 | 2.5 | const | -4.5 | -5.0 | -4.0 |
|  |  |  |  | PI-RADS | 0.87 | 0.74 | 1.00 |
|  |  |  |  | Output of Model 4 | 2.9 | 2.2 | 3.6 |
| MGH | Model 5 | -1.7 | 2.2 | const | -4.2 | -4.6 | -3.8 |
|  |  |  |  | PI-RADS | 1.0 | 0.92 | 1.1 |
|  |  |  |  | RSIrs-max | 0 | 0 | 0 |
| MGH | Model 6 | -1.7 | 2.4 | const | -4.6 | -5.0 | -4.2 |
|  |  |  |  | PI-RADS | 0.89 | 0.79 | 0.99 |
|  |  |  |  | Output of Model 3 | 2.6 | 2.1 | 3.1 |
| MGH | Model 7 | -1.7 | 2.5 | const | -4.5 | -4.9 | -4.1 |
|  |  |  |  | PI-RADS | 0.85 | 0.75 | 0.95 |
|  |  |  |  | Output of Model 4 | 3.0 | 2.5 | 3.6 |
| UCSF | Model 5 | -1.7 | 2.2 | const | -4.3 | -4.7 | -3.9 |
|  |  |  |  | PI-RADS | 1.0 | 0.93 | 1.1 |
|  |  |  |  | RSIrs-max | 0 | 0 | 0 |
| UCSF | Model 6 | -1.8 | 2.5 | const | -4.7 | -5.1 | -4.2 |
|  |  |  |  | PI-RADS | 0.91 | 0.81 | 1.0 |
|  |  |  |  | Output of Model 3 | 2.5 | 2.0 | 3.1 |
| UCSF | Model 7 | -1.8 | 2.6 | const | -4.6 | -5.0 | -4.2 |
|  |  |  |  | PI-RADS | 0.87 | 0.77 | 0.98 |
|  |  |  |  | Output of Model 4 | 3.0 | 2.4 | 3.5 |
| UTHSCSA | Model 5 | -1.7 | 2.2 | const | -4.12 | -4.6 | -3.8 |
|  |  |  |  | PI-RADS | 1.0 | 0.95 | 1.2 |
|  |  |  |  | RSIrs-max | 0 | 0 | 0 |
| UTHSCSA | Model 6 | -1.7 | 2.5 | const | -4.6 | -5.0 | -4.2 |
|  |  |  |  | PI-RADS | 0.91 | 0.80 | 1.0 |
|  |  |  |  | Output of Model 3 | 2.5 | 2.0 | 3.1 |
| UTHSCSA | Model 7 | -1.7 | 2.6 | const | -4.5 | -4.9 | -4.1 |
|  |  |  |  | PI-RADS | 0.854 | 0.748 | 0.959 |
|  |  |  |  | Output of Model 4 | 3.169 | 2.564 | 3.774 |
| URMC | Model 5 | -1.7 | 2.3 | const | -4.195 | -4.618 | -3.773 |
|  |  |  |  | PI-RADS | 1.028 | 0.923 | 1.134 |
|  |  |  |  | RSIrs-max | 0.001 | 0 | 0.002 |
| URMC | Model 6 | -1.9 | 2.7 | const | -4.961 | -5.432 | -4.49 |
|  |  |  |  | PI-RADS | 0.883 | 0.777 | 0.989 |
|  |  |  |  | Output of Model 3 | 3.287 | 2.648 | 3.926 |
| URMC | Model 7 | -1.9 | 2.7 | const | -4.718 | -5.166 | -4.271 |
|  |  |  |  | PI-RADS | 0.851 | 0.744 | 0.958 |
|  |  |  |  | Output of Model 4 | 3.387 | 2.752 | 4.021 |

Supplementary Table 2. Logistic Regression coefficients, 95% CIs, calibration slope and intercept for the combined model (Model 5, 6 and 7). Model **3** refers to the 3D DenseNet with T2w, ADC and high-b DWI input (bpMRI); Model **4** refers to the 3D-DenseNet+RSI with bpMRI, RSI-C_1_, RSI-C_2_ (RSI) and RSIrs-max input; Model **5** refers to the logistic regression model with PI-RADS and RSIrs-max input; Model **6** refers to the logistic regression model with PI-RADS and the output probability of Model **3**; Model **7** refers to the logistic regression model with PI-RADS and the output probability of Model **4**.

| **Institution** | **Scanner models** | **Number of Stations** |
| --- | --- | --- |
| UCSD Health | GE Healthcare Discovery MR750, GE Healthcare Signa Premier | 4 |
| UCSD CTIPM | GE Healthcare Discovery MR750, GE Healthcare Signa Premier | 4 |
| MGH | GE Healthcare Signa Premier | 1 |
| URMC | SIEMENS Magnetom Skyra | 2 |
| UCSF | GE Healthcare Signa Premier | 2 |
| UTHSCSA | SIEMENS Magnetom Skyra, SIEMENS Magnetom Trio | 3 |
| Cambridge | GE Healthcare Discovery MR750 | 1 |
| **Total** | 4 scanner models | 17 stations |

Supplementary Table 3. Scanner models and number of stations for each cohort. UCSD Health = University of California San Diego Health. UCSD CTIPM = The Center for Translational Imaging and Precision Medicine at the University of California San Diego. MGH = Harvard University affiliated Massachusetts General Hospital. URMC = University of Rochester Medical Center. UCSF = University of California San Francisco. UTHSCSA = University of Texas Health Sciences Center San Antonio. Cambridge = University of Cambridge.

| **UCSD Health** | **RSI** | **DWI** | ***T_2_*-weighted** |
| --- | --- | --- | --- |
| Pulse sequence | Diffusion-weighted EPI | Diffusion-weighted EPI | Fast Spin Echo (FSE) |
| TR (ms) | 4000 | 4500 | 5300 |
| TE (ms) | 69 | 55 | 100 |
| FOV (mm) | 240 x 120 | 180 x 90 | 200 x 200 |
| Matrix [resampled dimensions] | 96 x 48 [256 x 256] | 112 x 56 [256 x 256] | 320 x 320 [512 x 512] |
| Slices | 16 | 34 | 32 |
| Slice Thickness (mm) | 6 | 3 | 3 |
| b-values (s/mm^2^) [number of samples] | 0 [1], 500 [8], 1000 [8], 2000 [16] | 50 [6], 1400 [18] | N/A |
| Field Strength (T) | 3 | 3 | 3 |
| **UCSD CTIPM** | **RSI** | **DWI** | ***T_2_*-weighted** |
| Pulse sequence | Diffusion-weighted EPI | Diffusion-weighted EPI | Fast Spin Echo (FSE) |
| TR (ms) | 4500 | 4500 | 7000 |
| TE (ms) | 69 | 51 | 100 |
| FOV (mm) | 240 x 120 | 160 x 88 | 240 x 240 |
| Matrix [resampled dimensions] | 96 x 48 [128 x 128] | 100 x 50 [256 x 256] | 320 x 320 [512 x 512] |
| Slices | 16 | 32 | 32 |
| Slice Thickness (mm) | 6 | 3 | 3 |
| b-values (s/mm^2^) [number of samples] | 0 [1], 500 [6], 1000 [6], 2000 [12] | 50 [6], 1000 [18] | N/A |
| Field Strength (T) | 3 | 3 | 3 |
| **MGH** | **RSI** | **DWI** | ***T_2_*-weighted** |
| Pulse sequence | Diffusion-weighted EPI | Diffusion-weighted EPI | Fast Spin Echo (FSE) |
| TR (ms) | 4500 | 4777 | 3937 |
| TE (ms) | 59 | 54 | 169 |
| FOV (mm) | 240 x 120 | 320 x 320 | 160 x 160 |
| Matrix [resampled dimensions] | 96 x 48 [128x128] | 128 x 128 [256 x 256] | 360 x 224 [1024 x 1024] |
| Slices | 16 | 45 | 40 |
| Slice Thickness (mm) | 6 | 3 | 3 |
| b-values (s/mm^2^) [number of samples] | 0 [1], 500 [6], 1000 [6], 2000 [12] | 50 [1], 800 [1] | N/A |
| Field Strength (T) | 3 | 3 | 3 |
| **URMC** | **RSI** | **DWI** | ***T_2_*-weighted** |
| Pulse sequence | Diffusion-weighted EPI | Diffusion-weighted EPI | Fast Spin Echo (FSE) |
| TR (ms) | 3800 | 4900 | 4800 |
| TE (ms) | 85 | 89 | 104 |
| FOV (mm) | 52 x 52 | 249 x 249 | 180x180 |
| Matrix [resampled dimensions] | 100 x 52 [104 x 200] | 114 x 114 [114 x 114] | 384 x 365 [384 x 384] |
| Slices | 22 | 66 | 32 |
| Slice Thickness (mm) | 4 | 3.5 | 3 |
| b-values (s/mm^2^) [number of samples] | 0 [1], 500 [6], 1000 [6], 2000 [6] | 50 [1], 400 [1], 800 [1] | N/A |
| Field Strength (T) | 3 | 3 | 3 |
| **UCSF** | **RSI** | **DWI** | ***T_2_*-weighted** |
| Pulse sequence | Diffusion-weighted EPI | Diffusion-weighted EPI | Fast Spin Echo (FSE) |
| TR (ms) | 4500 | 4800 | 2964 |
| TE (ms) | 73 | 46 | 150 |
| FOV (mm) | 200 x 200 | 200 x 100 | 220 x 220 |
| Matrix [resampled dimensions] | 256 x 256 [256 x 256] | 128 x 64 [256 x 256] | 512 x 512 [320 x 320] |
| Slices | 35 | 37 | 36 |
| Slice Thickness (mm) | 3 | 3 | 3 |
| b-values (s/mm^2^) [number of samples] | 0 [5], 100 [6], 800 [12], 1400 [12], 2500 [18] | 0 [1], 600 [1], 1000 [3] | N/A |
| Field Strength (T) | 3 | 3 | 3 |
| **UTHSCSA** | **RSI** | **DWI** | ***T_2_*-weighted** |
| Pulse sequence | Diffusion-weighted EPI | Diffusion-weighted EPI | Fast Spin Echo (FSE) |
| TR (ms) | 6300 | 5500 | 4710 |
| TE (ms) | 105 | 88 | 100 |
| FOV (mm) | 52 x 100 | 115 x 115 | 180 x 180 |
| Matrix [resampled dimensions] | 52 x 100 [104 x 200] | 160 x 133 [160 x 160] | 240 x 320 [320 x 320] |
| Slices | 22 | 25 | 30 |
| Slice Thickness (mm) | 4 | 3 | 3 |
| b-values (s/mm^2^) [number of samples] | 0 [1], 500 [6], 1000 [18], 2000 [42] | 0 [3], 400 [18], 800 [18], 1000 [18] | N/A |
| Field Strength (T) | 3 | 3 | 3 |
| **Cambridge** | **RSI** | **DWI** | ***T_2_*-weighted** |
| Pulse sequence | Diffusion-weighted EPI | N/A | Fast Spin Echo (FSE) |
| TR (ms) | 4500 | N/A | 3130 |
| TE (ms) | 68 | N/A | 98 |
| FOV (mm) | 220 x 110 | N/A | 180 x 180 |
| Matrix [resampled dimensions] | 96 x 48 [256 x 256] | N/A | 448 x 256 [512 x 512] |
| Slices | 8 | N/A | 30 |
| Slice Thickness (mm) | 4 | N/A | 3 |
| b-values (s/mm^2^) [number of samples] | 0 [1], 500 [2], 1000 [2], 2000 [4] | N/A | N/A |
| Field Strength (T) | 3 | N/A | 3 |

Supplementary Table 4. MRI acquisition parameters for each cohort. UCSD Health = University of California San Diego Health. UCSD CTIPM = The Center for Translational Imaging and Precision Medicine at the University of California San Diego. MGH = Harvard University affiliated Massachusetts General Hospital. URMC = University of Rochester Medical Center. UCSF = University of California San Francisco. UTHSCSA = University of Texas Health Sciences Center San Antonio. Cambridge = University of Cambridge. TR = repetition time. TE = echo time. FOV = field-of-view. FSE = fast spin echo. EPI = echo-planar imaging. RSI = Restriction Spectrum Imaging. DWI = diffusion-weighted imaging.

| UCSD Health | AUC | Threshold@Fixed Sensitivity = 0.90 | Specificity@Fixed Sensitivity = 0.90 | ΔSpecificity@Fixed Sensitivity = 0.90 | PPV@Fixed Sensitivity = 0.90 | NPV@Fixed Sensitivity = 0.90 | Accuracy@Fixed Sensitivity = 0.90 | NRI | IDI |
| --- | --- | --- | --- | --- | --- | --- | --- | --- | --- |
| Model 1 | 0.74 (0.68-0.79) | PI-RADS 4 (4-4) | 0.42 (0.32-0.51) | 0 (0-0) | 0.71 (0.65-0.77) | 0.75 (0.63-0.85) | 0.72 (0.67-0.77) | 0 (0-0) | 0 (0-0) |
| Model 2 | 0.75 (0.69-0.80) | 215.57 (191.30-236.54) | 0.32 (0.20-0.49) | -0.09 (-0.09--0.09) | 0.68 (0.61-0.75) | 0.67 (0.55-0.76) | 0.68 (0.62-0.75) | 0.07 (0.07-0.07) | -0.21 (-0.21--0.21) |
| Model 3 | 0.78 (0.72-0.83) | 0.42 (0.38-0.48) | 0.47 (0.34-0.61) | 0.06 (0.05-0.06) | 0.73 (0.66-0.80) | 0.75 (0.66-0.81) | 0.74 (0.68-0.79) | 0.12 (0.12-0.12) | 0.02 (0.02-0.02) |
| Model 4 | 0.80 (0.75-0.85) | 0.26 (0.20-0.30) | 0.48 (0.33-0.61) | 0.06 (0.06-0.07) | 0.74 (0.66-0.80) | 0.75 (0.66-0.81) | 0.74 (0.68-0.79) | 0.13 (0.13-0.13) | 0.04 (0.04-0.04) |
| Model 5 | 0.79 (0.74-0.84) | 0.51 (0.32-0.52) | 0.46 (0.34-0.58) | 0.04 (0.04-0.04) | 0.73 (0.66-0.79) | 0.74 (0.66-0.80) | 0.73 (0.68-0.78) | 0.16 (0.16-0.16) | -0.04 (-0.04--0.04) |
| Model 6 | 0.80 (0.74-0.85) | 0.49 (0.35-0.54) | 0.53 (0.34-0.67) | 0.11 (0.10-0.11) | 0.75 (0.67-0.82) | 0.77 (0.67-0.82) | 0.76 (0.68-0.81) | 0.16 (0.16-0.17) | 0.03 (0.03-0.03) |
| Model 7 | 0.82 (0.76-0.86) | 0.36 (0.31-0.42) | 0.52 (0.37-0.68) | 0.10 (0.10-0.11) | 0.75 (0.68-0.83) | 0.77 (0.68-0.82) | 0.75 (0.69-0.82) | 0.19 (0.19-0.19) | 0.05 (0.05-0.05) |
| UCSD CTIPM | AUC | Threshold@Fixed Sensitivity = 0.90 | Specificity@Fixed Sensitivity = 0.90 | ΔSpecificity@Fixed Sensitivity = 0.90 | PPV@Fixed Sensitivity = 0.90 | NPV@Fixed Sensitivity = 0.90 | Accuracy@Fixed Sensitivity = 0.90 | NRI | IDI |
| Model 1 | 0.74 (0.68-0.79) | PI-RADS 4 (3-4) | 0.41 (0.12-0.52) | 0 (0-0) | 0.64 (0.53-0.72) | 0.75 (0.52-0.85) | 0.67 (0.54-0.73) | 0 (0-0) | 0 (0-0) |
| Model 2 | 0.75 (0.69-0.81) | 170.17 (150.24-189.43) | 0.28 (0.13-0.50) | -0.07 (-0.07--0.06) | 0.61 (0.52-0.70) | 0.70 (0.51-0.82) | 0.62 (0.54-0.72) | 0.04 (0.04-0.04) | -0.20 (-0.20--0.20) |
| Model 3 | 0.77 (0.71-0.82) | 0.30 (0.24-0.35) | 0.43 (0.28-0.56) | 0.07 (0.06-0.07) | 0.66 (0.58-0.73) | 0.78 (0.69-0.84) | 0.69 (0.61-0.75) | 0.05 (0.05-0.05) | 0 (0-0) |
| Model 4 | 0.77 (0.71-0.82) | 0.30 (0.28-0.36) | 0.37 (0.27-0.55) | 0.02 (0.01-0.02) | 0.64 (0.57-0.72) | 0.75 (0.67-0.83) | 0.66 (0.60-0.74) | 0.05 (0.05-0.06) | 0.01 (0.01-0.01) |
| Model 5 | 0.79 (0.73-0.84) | 0.37 (0.25-0.39) | 0.45 (0.31-0.58) | 0.09 (0.09-0.09) | 0.67 (0.59-0.74) | 0.79 (0.70-0.84) | 0.70 (0.63-0.76) | 0.07 (0.07-0.07) | 0.05 (0.05-0.05) |
| Model 6 | 0.79 (0.73-0.84) | 0.33 (0.18-0.44) | 0.44 (0.19-0.65) | 0.08 (0.08-0.09) | 0.67 (0.56-0.77) | 0.78 (0.61-0.85) | 0.70 (0.58-0.79) | 0.10 (0.10-0.10) | 0.03 (0.03-0.03) |
| Model 7 | 0.79 (0.74-0.85) | 0.42 (0.30-0.47) | 0.47 (0.25-0.64) | 0.10 (0.10-0.10) | 0.67 (0.57-0.77) | 0.79 (0.66-0.86) | 0.71 (0.60-0.78) | 0.10 (0.10-0.10) | 0.04 (0.04-0.04) |
| MGH | AUC | Threshold@Fixed Sensitivity = 0.90 | Specificity@Fixed Sensitivity = 0.90 | ΔSpecificity@Fixed Sensitivity = 0.90 | PPV@Fixed Sensitivity = 0.90 | NPV@Fixed Sensitivity = 0.90 | Accuracy@Fixed Sensitivity = 0.90 | NRI | IDI |
| Model 1 | 0.77 (0.61-0.91) | PI-RADS 4 (3-4) | 0.50 (0-0.83) | 0 (0-0) | 0.62 (0.37-0.85) | 0.83 (0.50-1.00) | 0.69 (0.39-0.87) | 0 (0-0) | 0 (0-0) |
| Model 2 | 0.85 (0.70-0.96) | 240.75 (213.25-295.00) | 0.54 (0.18-1.00) | 0.10 (0.10-0.11) | 0.66 (0.42-1.00) | 0.86 (0.57-1.00) | 0.72 (0.50-0.94) | 0.17 (0.17-0.17) | -0.32 (-0.32--0.31) |
| Model 3 | 0.75 (0.57-0.91) | 0.52 (0.44-0.62) | 0.55 (0.20-0.89) | 0.09 (0.09-0.10) | 0.65 (0.43-0.89) | 0.86 (0.62-1.00) | 0.72 (0.52-0.89) | 0.06 (0.06-0.07) | -0.18 (-0.18--0.18) |
| Model 4 | 0.77 (0.59-0.91) | 0.35 (0.32-0.47) | 0.45 (0.12-0.86) | 0.01 (0-0.01) | 0.61 (0.39-0.86) | 0.83 (0.50-1.00) | 0.67 (0.46-0.88) | 0.03 (0.03-0.03) | -0.17 (-0.17--0.17) |
| Model 5 | 0.83 (0.66-0.96) | 0.32 (0.05-0.58) | 0.75 (0.05-1.00) | 0.19 (0.18-0.19) | 0.76 (0.40-1.00) | 0.88 (0.33-1.00) | 0.82 (0.43-0.96) | 0.24 (0.24-0.24) | -0.05 (-0.05--0.05) |
| Model 6 | 0.83 (0.67-0.95) | 0.40 (0.09-0.64) | 0.58 (0.12-0.94) | 0.09 (0.09-0.09) | 0.66 (0.40-0.93) | 0.86 (0.50-1.00) | 0.73 (0.47-0.92) | 0.12 (0.12-0.12) | -0.04 (-0.04--0.04) |
| Model 7 | 0.85 (0.69-0.96) | 0.35 (0.10-0.65) | 0.56 (0.12-1.00) | 0.09 (0.09-0.10) | 0.66 (0.40-1.00) | 0.86 (0.50-1.00) | 0.72 (0.47-0.94) | 0.16 (0.16-0.16) | -0.03 (-0.03--0.02) |
| URMC | AUC | Threshold@Fixed Sensitivity = 0.90 | Specificity@Fixed Sensitivity = 0.90 | ΔSpecificity@Fixed Sensitivity = 0.90 | PPV@Fixed Sensitivity = 0.90 | NPV@Fixed Sensitivity = 0.90 | Accuracy@Fixed Sensitivity = 0.90 | NRI | IDI |
| Model 1 | 0.75 (0.69-0.80) | PI-RADS 3 (3-3) | 0.18 (0.12-0.25) | 0 (0-0) | 0.50 (0.43-0.57) | 0.86 (0.70-0.97) | 0.54 (0.48-0.61) | 0 (0-0) | 0 (0-0) |
| Model 2 | 0.70 (0.64-0.77) | 259.34 (228.76-282.58) | 0.42 (0.24-0.56) | 0.23 (0.23-0.23) | 0.57 (0.48-0.66) | 0.83 (0.73-0.88) | 0.64 (0.54-0.72) | -0.03 (-0.03--0.03) | -0.25 (-0.25--0.25) |
| Model 3 | 0.71 (0.64-0.78) | 0.09 (0.07-0.16) | 0.37 (0.23-0.57) | 0.20 (0.20-0.20) | 0.55 (0.47-0.66) | 0.81 (0.72-0.88) | 0.62 (0.53-0.72) | 0 (0-0) | -0.05 (-0.05--0.05) |
| Model 4 | 0.73 (0.67-0.80) | 0.14 (0.09-0.20) | 0.40 (0.20-0.58) | 0.21 (0.21-0.21) | 0.56 (0.46-0.66) | 0.82 (0.69-0.88) | 0.63 (0.51-0.73) | 0.02 (0.02-0.02) | -0.05 (-0.05--0.05) |
| Model 5 | 0.77 (0.71-0.83) | 0.31 (0.30-0.32) | 0.47 (0.27-0.60) | 0.27 (0.27-0.27) | 0.59 (0.48-0.68) | 0.84 (0.74-0.89) | 0.67 (0.55-0.74) | 0.05 (0.05-0.05) | -0.03 (-0.03--0.03) |
| Model 6 | 0.77 (0.71-0.82) | 0.14 (0.12-0.18) | 0.43 (0.26-0.57) | 0.24 (0.24-0.24) | 0.57 (0.48-0.66) | 0.83 (0.74-0.88) | 0.65 (0.55-0.72) | 0.07 (0.07-0.07) | 0.01 (0.01-0.01) |
| Model 7 | 0.78 (0.72-0.84) | 0.18 (0.14-0.25) | 0.45 (0.27-0.62) | 0.26 (0.26-0.26) | 0.58 (0.48-0.68) | 0.84 (0.75-0.89) | 0.66 (0.55-0.75) | 0.08 (0.08-0.08) | 0.02 (0.02-0.02) |
| UCSF | AUC | Threshold@Fixed Sensitivity = 0.90 | Specificity@Fixed Sensitivity = 0.90 | ΔSpecificity@Fixed Sensitivity = 0.90 | PPV@Fixed Sensitivity = 0.90 | NPV@Fixed Sensitivity = 0.90 | Accuracy@Fixed Sensitivity = 0.90 | NRI | IDI |
| Model 1 | 0.66 (0.41-0.87) | PI-RADS 4 (4-5) | 0.11 (0-0.62) | 0 (0-0) | 0.64 (0.41-0.88) | 1.00 (0.83-1.00) | 0.65 (0.43-0.87) | 0 (0-0) | 0 (0-0) |
| Model 2 | 0.59 (0.29-0.86) | 123.42 (117.97-273.77) | 0.29 (0-0.83) | 0.17 (0.16-0.17) | 0.67 (0.44-0.92) | 0.67 (0-1.00) | 0.67 (0.47-0.88) | 0.09 (0.09-0.10) | -0.14 (-0.14--0.14) |
| Model 3 | 0.77 (0.50-0.96) | 0.36 (0.29-0.46) | 0.38 (0-1.00) | 0.30 (0.30-0.31) | 0.73 (0.46-1.00) | 0.80 (0-1.00) | 0.73 (0.50-0.94) | 0.31 (0.30-0.31) | 0.06 (0.06-0.06) |
| Model 4 | 0.90 (0.69-1.00) | 0.55 (0.38-0.56) | 0.83 (0.25-1.00) | 0.65 (0.65-0.66) | 0.90 (0.61-1.00) | 0.86 (0.60-1.00) | 0.88 (0.67-1.00) | 0.47 (0.46-0.47) | 0.15 (0.14-0.14) |
| Model 5 | 0.66 (0.36-0.91) | 0.50 (0.50-0.68) | 0.40 (0-0.86) | 0.27 (0.26-0.27) | 0.71 (0.46-0.93) | 0.75 (0.25-1.00) | 0.71 (0.50-0.90) | 0.15 (0.15-0.15) | -0.03 (-0.03--0.02) |
| Model 6 | 0.78 (0.51-0.96) | 0.48 (0.44-0.69) | 0.50 (0-1.00) | 0.35 (0.34-0.35) | 0.74 (0.50-1.00) | 0.80 (0.33-1.00) | 0.75 (0.53-0.94) | 0.29 (0.29-0.29) | 0.05 (0.05-0.05) |
| Model 7 | 0.86 (0.62-1.00) | 0.57 (0.51-0.70) | 0.75 (0.25-1.00) | 0.59 (0.58-0.59) | 0.86 (0.57-1.00) | 0.86 (0.50-1.00) | 0.84 (0.65-1.00) | 0.40 (0.40-0.40) | 0.09 (0.09-0.10) |
| UTHSCSA | AUC | Threshold@Fixed Sensitivity = 0.90 | Specificity@Fixed Sensitivity = 0.90 | ΔSpecificity@Fixed Sensitivity = 0.90 | PPV@Fixed Sensitivity = 0.90 | NPV@Fixed Sensitivity = 0.90 | Accuracy@Fixed Sensitivity = 0.90 | NRI | IDI |
| Model 1 | 0.64 (0.36-0.90) | PI-RADS 4 (4-5) | 0.33 (0-0.80) | 0 (0-0) | 0.62 (0.33-0.89) | 0.80 (0-1.00) | 0.65 (0.38-0.88) | 0 (0-0) | 0 (0-0) |
| Model 2 | 0.85 (0.58-1.00) | 219.26 (208.13-358.95) | 0.60 (0.12-1.00) | 0.29 (0.29-0.30) | 0.75 (0.43-1.00) | 0.88 (0.50-1.00) | 0.78 (0.53-1.00) | 0.37 (0.37-0.38) | -0.09 (-0.09--0.09) |
| Model 3 | 0.88 (0.62-1.00) | 0.47 (0.24-0.73) | 0.83 (0-1.00) | 0.38 (0.38-0.39) | 0.89 (0.40-1.00) | 0.89 (0.60-1.00) | 0.87 (0.44-1.00) | 0.47 (0.46-0.47) | 0.20 (0.19-0.20) |
| Model 4 | 0.90 (0.67-1.00) | 0.51 (0.02-0.74) | 1.00 (0-1.00) | 0.48 (0.47-0.49) | 1.00 (0.41-1.00) | 0.90 (0.67-1.00) | 0.92 (0.44-1.00) | 0.51 (0.51-0.52) | 0.29 (0.29-0.29) |
| Model 5 | 0.78 (0.48-1.00) | 0.55 (0.31-0.80) | 0.50 (0-1.00) | 0.19 (0.18-0.19) | 0.69 (0.40-1.00) | 0.86 (0.50-1.00) | 0.73 (0.48-0.95) | 0.28 (0.28-0.28) | 0.02 (0.02-0.02) |
| Model 6 | 0.79 (0.50-1.00) | 0.42 (0.34-0.83) | 0.50 (0-1.00) | 0.19 (0.19-0.19) | 0.69 (0.38-1.00) | 0.86 (0.50-1.00) | 0.73 (0.47-0.96) | 0.29 (0.29-0.29) | 0.11 (0.11-0.11) |
| Model 7 | 0.85 (0.58-1.00) | 0.41 (0.28-0.88) | 0.58 (0.12-1.00) | 0.29 (0.28-0.29) | 0.75 (0.43-1.00) | 0.88 (0.50-1.00) | 0.77 (0.53-1.00) | 0.37 (0.37-0.38) | 0.16 (0.16-0.16) |

Supplementary Table 5. AUCs, Threshold of each model at fixed sensitivity of 0.90, Specificity of each model at fixed sensitivity of 0.90, ΔSpecificity of each model at fixed sensitivity of 0.90, Positive Predictive Value (PPV) at fixed sensitivity of 0.90, Negative Predictive Value (NPV) at fixed sensitivity of 0.90, Accuracy at fixed sensitivity of 0.90, Net Reclassification Improvement (NRI), Integrated Discrimination Improvement (IDI) for each cohort. The result is shown in median and 95 percent confidence interval in the parentheses. UCSD Health = University of California San Diego Health. UCSD CTIPM = The Center for Translational Imaging and Precision Medicine at the University of California San Diego. MGH = Harvard University affiliated Massachusetts General Hospital. Model 1 refers to the logistic regression model with PI-RADS input; Model 2 refers to the logistic regression model with RSIrs-max input; Model 3 refers to the 3D DenseNet with T2w, ADC and high-b DWI input (bpMRI); Model 4 refers to the 3D-DenseNet+RSI with bpMRI, RSI-C_1_, RSI-C_2_ (RSI) and RSIrs-max input; Model 5 refers to the logistic regression model with PI-RADS and RSIrs-max input; Model 6 refers to the logistic regression model with PI-RADS and the output probability of Model 3; Model 7 refers to the logistic regression model with PI-RADS and the output probability of Model 4.
